# In Situ Burning of Oil Spills

**DOI:** 10.6028/jres.106.009

**Published:** 2001-02-01

**Authors:** David D. Evans, George W. Mulholland, Howard R. Baum, William D. Walton, Kevin B. McGrattan

**Affiliations:** National Institute of Standards and Technology, Gaithersburg, MD 20899-8640

**Keywords:** ALOFT, combustion, large eddy simulation, offshore drilling, oil spills, pool fires, smoke plumes, smoke sampling, smoke yield

## Abstract

For more than a decade NIST conducted research to understand, measure and predict the important features of burning oil on water. Results of that research have been included in nationally recognized guidelines for approval of intentional burning. NIST measurements and predictions have played a major role in establishing in situ burning as a primary oil spill response method. Data are given for pool fire burning rates, smoke yield, smoke particulate size distribution, smoke aging, and polycyclic aromatic hydrocarbon content of the smoke for crude and fuel oil fires with effective diameters up to 17.2 m. New user-friendly software, ALOFT, was developed to quantify the large-scale features and trajectory of wind blown smoke plumes in the atmosphere and estimate the ground level smoke particulate concentrations. Predictions using the model were tested successfully against data from large-scale tests. ALOFT software is being used by oil spill response teams to help assess the potential impact of intentional burning.

## 1. Introduction

One of the risks of oil drilling and transportation is that accidents can occur releasing natural crude oil or its refined products in oil spills. Oil contamination of land or water is an environmental hazard to life. Historically oil spill response has been limited to various mechanical means of recovering the spilled oil from land or water and then disposing of or reprocessing the waste. Generally large amounts of oil contaminated materials need to be removed and treated. Mechanical recovery of oil in areas such as rocky shorelines, marshlands, and in ice-laden waterways is impractical. Industry needs to have alternative technologies to mechanical recovery for oil spill response. One of the possible alternatives is to burn the oil in place—in situ burning.

The 1989 oil spill from the *Exxon Valdez* tanker onto the waters of Prince Williams Sound in Alaska focused national attention on oil spills. An estimated 42 million liters of oil were released from the ship into the water. Some of the oil, driven by winds and currents, was deposited on the shoreline of Prince Williams Sound. At the time of that spill, NIST and others were already engaged in the evaluation of burning as a response to oil spills. Industry was beginning to produce fire resistant booms that could be used to confine oil spilled on water in order to burn it in place. It is a little known fact that using a fire resistant boom, approximately 57 000 L of oil from the *Exxon Valdez* that had been in the water for nearly two days was confined and burned. The resulting fire lasting approximately 45 min consumed all but 1100 L of residue that remained in the boom [1].

Any response to oil spills includes considerations of oil containment, recovery, disposal and the logistics of delivering adequate response equipment quickly to the spill site. Particularly in remote areas, the use of burning as a oil spill response method is attractive. Burning requires a minimum of equipment, and because the oil is gasified during combustion, the need for physical collection, storage, and transport of recovered product is reduced to the few percent of the original spill volume that remains as residue after burning.

Oil spilled on water begins to spread naturally away from the source. Wind, waves and currents move the oil over the water surface and also contribute to emulsification. Thin layers of oil on water cannot be burned. So in order to ignite and sustain the burning of oil spills, the oil needs to be confined. In some cases natural confinement such as ice leads provides the confinement. In general, responders need to provide a means to thicken the oil layer and confine the burning. To do this, artificial confinement is needed within fire resistant booms.

Burning oil spills in-place normally produces a visible smoke plume containing soot and other combustion products produced in the burning. Lack of knowledge about the extent of the area affected by the smoke plume produced by burning of crude oil spills and the possibility of undesirable combustion products carried in the plume have led to public concerns over the effects of intentional burning large crude oil spills. Unresolved questions about personnel and equipment safety from the heat and thermal radiation produced by large fires has also hampered application of burning to oil spills.

In the decision process for approval of intentional burning of oil spills, local authorities need to have tools to quantify the likely benefits of the burning in terms of oil removal and the likely consequences in terms of the fire generated smoke plume. The in situ oil spill research program at the National Institute of Standards and Technology (NIST) was designed to develop quantitative information and software tools to aid authorities in making informed decisions. The lack of this information was seen as an impediment to the acceptance and use of this emerging technology.

In order to do this NIST designed a comprehensive program of integrated measurements and predictions. Understanding the process of burning oil on water and its consequences involved fire experiments in NIST laboratories, in fire research facilities in Japan, at new field facilities specifically constructed for this research in Mobile, Alabama, and at large scale oil burn experiments in Alaska and offshore Newfoundland, Canada. New measurement instruments were developed to implement successful laboratory techniques into robust air-deployable self-powered measurement and sampling packages. New computational methods for fire driven buoyant flows were exploited to quantify wind driven smoke trajectories in the atmosphere and estimate the downwind particulate concentrations. Finally, user friendly software was developed to provide the benefits of this research to emergency responders and local authorities.

Burning may be thought of as an emerging technology for response to oil spills. NIST has been a major contributor to the science and technology used for safe and effective in situ burning of oil spills. A major factor aiding the NIST research effort has been the relationships built up over the years with a number of organizations. Since 1985, NIST has enjoyed long term and substantial funding from the Minerals Management Service, U.S. Department of the Interior. NIST has also worked closely with partners including the United States Coast Guard, U.S. Department of Transportation, the Technology Development and Technical Services Branch of Environment Canada, the Environmental Response Team of the U.S. Environmental Protection Agency, Alaska Clean Seas (an oil industry cooperative), and the National Research Institute for Fire and Disaster in Japan.

## 2. Background

The in situ burning of oil spills has historically been regarded as a response method of last resort. The dynamics of ignition and sustained burning of oil spills has not been understood. An early attempt to analyze the process of oil spill burning and to set down guidelines for when it would and would not be successful was the work of Thompson et al. in 1979 [2]. Prior to that work, testing had been performed largely for the demonstration of various products being developed to ignite and promote the burning of spills. Thompson reviewed the use of in situ burning in response to spill accidents. His review provides a perspective on the technology in use in the 1970s and the mixed results in practice responding to major oil spill incidents. In general since burning was regarded as a response method of last resort, oil could have been in the water for days before burning was attempted. The longer oil stays in the water allowing volatile components to evaporate (weathering) and water to emulsify with the oil by wave action, the harder the spill is to ignite and burn. For in situ burning to be widely effective, it needed to be considered as one of the primary oil spill response methods.

In 1985, NIST began studies of oil spill burning on open waters and in channels formed by broken ice in support of the Safety in Offshore Drilling program of the Minerals Management Service, Department of the Interior. The original intent of the program was to find means to generalize the experimental results of Brown and Goodman [3] and Smith and Diaz [4] for the burning of oil in ice leads. Smith and Diaz burned oil on the water surface confined by ice blocks (simulating broken ice formations) in the EPA OHMSETT tank facility in Leonardo, NJ. The results of the experiments were encouraging with respect to the efficiency of in situ burning as typically better than 50 % of the oil spilled in the ice formation could be removed by burning and in some cases over 90 % was possible.

It was quickly realized that in order to gain acceptance for in situ burning of oil spill, being able to quantify the amount and effects of the smoke from the burn was of greater importance than quantifying the amount of oil removed from the water by burning. The latter was the focus of many of the studies funded by industry. To address the burning, smoke production, and smoke transport issues, it was clear that expertise in combustion, fire dynamics, computational fluid flow, particle measurement, chemical analysis, and large-scale fire measurements would be needed. NIST had the capabilities to assemble this interdisciplinary team and the facilities to support its experimental and computational effort.

As part of the NIST research program, in situ burning technologies were reviewed and input on research needs was gathered periodically through workshops conducted by NIST [5, 6, 7]. Other sources have also reviewed the technology of oil spill burning and other response methods [2, 8, 9].

## 3. Experimental Facilities

To understand and quantify the important features of in situ burning it was necessary to perform three scales of experiments. Laboratory tests furnished property data, experiments utilizing large-scale outdoor burn facilities provided mesoscale data and means to develop and evaluate instrumentation, and finally, actual burns of spilled oil at sea provided data on in situ burning at the anticipated scale of actual response operations. In this research program, there has been continued interaction between findings from measurements on small fire experiments performed in the controlled laboratory environments of NIST and the National Research Institute of Fire and Disaster (NRIFD) in Mitaka, Tokyo, Japan, and large fire experiments at facilities like the USCG Fire Safety and Test Detachment in Mobile, Alabama where outdoor liquid fuel burns in large pans are possible.

### 3.1 NIST Facilities

**Fig. 1:**
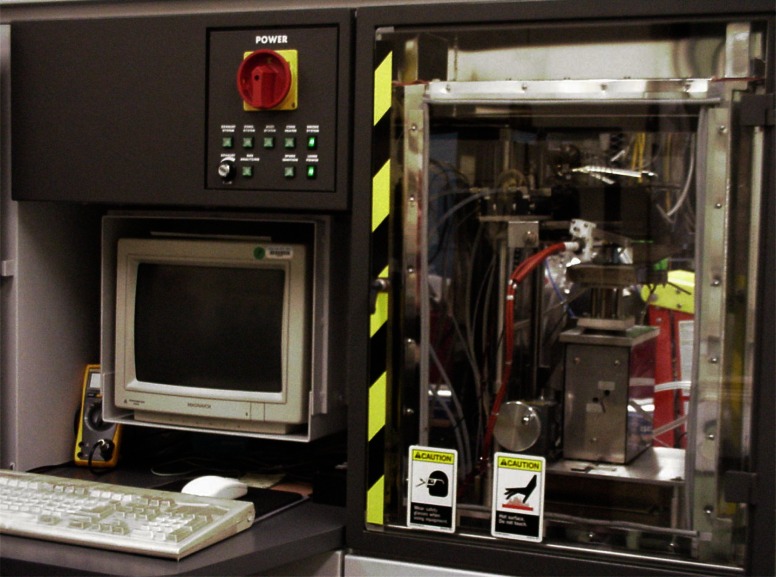
Cone calorimeter apparatus.

At NIST, two major facilities were used to perform measurements on crude oil pool fires ranging in size from 0.085 m to 0.6 m in diameter. The smallest fires, 0.085 m diameter, were conducted in the Cone Calorimeter to determine the effective heat of combustion for the crude oils and evaluate smoke yield using three different measurement methods. The Cone Calorimeter, shown in Fig. 1, is more formally known as Standard Test Method for Heat and Visible Smoke Release Rates for Materials and Products Using an Oxygen Consumption Calorimeter [10]. The name of the apparatus, Cone Calorimeter, is derived from the shape of the heater used to irradiate samples. The heater coils are formed along the inner surface of a truncated cone. By imposing additional thermal radiation on a small sample, the sample is made to burn as if it were in the interior of a larger fire. The major material flammability characteristics can be evaluated using this laboratory apparatus. These include: rate of heat release, effective heat of combustion, total heat release, ignitibility, mass loss rate, smoke specific extinction area, and yields of various gaseous species and particulate.

**Fig. 2:**
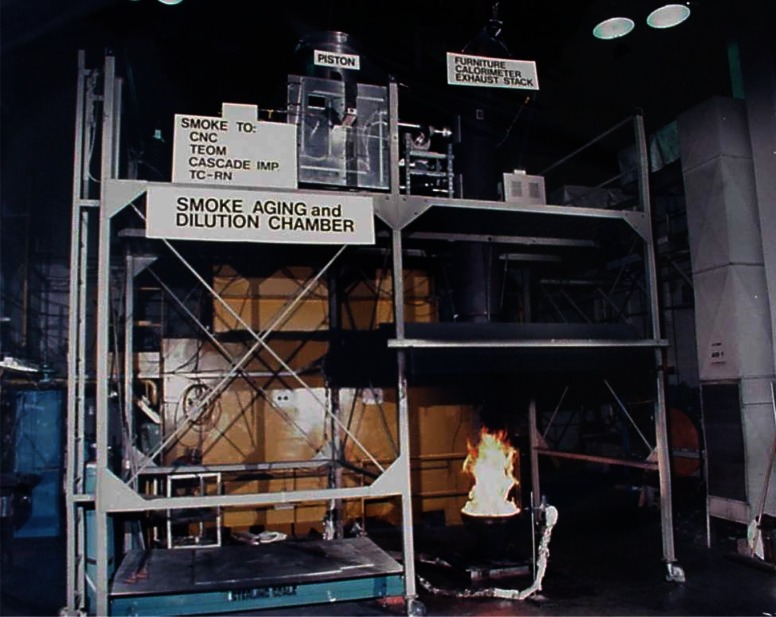
NIST large calorimeter with smoke sampling equipment installed.

A larger calorimeter apparatus capable of accommodating samples up to 0.6 m in diameter was used to provide NIST data about the amount and properties of smoke as the diameters of the fires increased. Extensive instrumentation and sampling hardware were added to the exhaust flow from the hood as shown in Fig. 2. Samples drawn from the exhaust hood duct were used to quantify the amount of each major combustion product generated per kilogram of crude oil burned, the chemical composition of the smoke including polycyclic aromatic hydrocarbon (PAH) content, the particulate size distribution of both fresh and aged smoke, and the oxygen consumed in the combustion process. Oxygen consumption calorimetry is used to measure the heat release rate of the fire, which is the primary quantity used to characterize burning intensity. To further characterize the combustion process, additional instrumentation was used to measure radiant heat flux from the flame and the mass loss rate of the burning fuel.

### 3.2 NRIFD Facilities

**Fig. 3:**
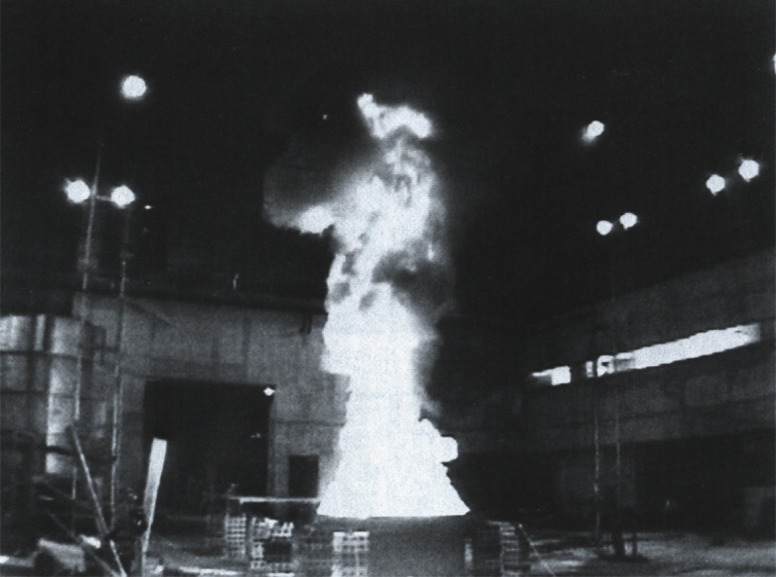
Two-meter diameter crude oil fire in NRIFD, Japan test hall.

The relatively small, 0.6 m diameter, fires provided a means of measuring fire characteristics under controlled conditions, but are too small to provide an adequate test of measurement equipment being developed for field use. Through the cooperation of NRIFD joint studies of crude oil burning characteristics were conducted. NRIFD maintains a fire test facility in which crude oil pools up to 3 m in diameter are burned, with all of the combustion products collected in a large hood system. Figure 3 shows a 2 m diameter crude oil fire burning in the 24 m × 24 m × 20 m high test hall. This facility could accommodate fires that are large enough so that sampling packages designed for mesoscale field tests could be evaluated by comparison to traditional laboratory measurements. To do this the exhaust system for the building was instrumented so that measurements similar to those performed in the NIST facility could be made by effectively using the entire NRIFD test building as a smoke collection hood.

### 3.3 USCG Facility

**Fig. 4:**
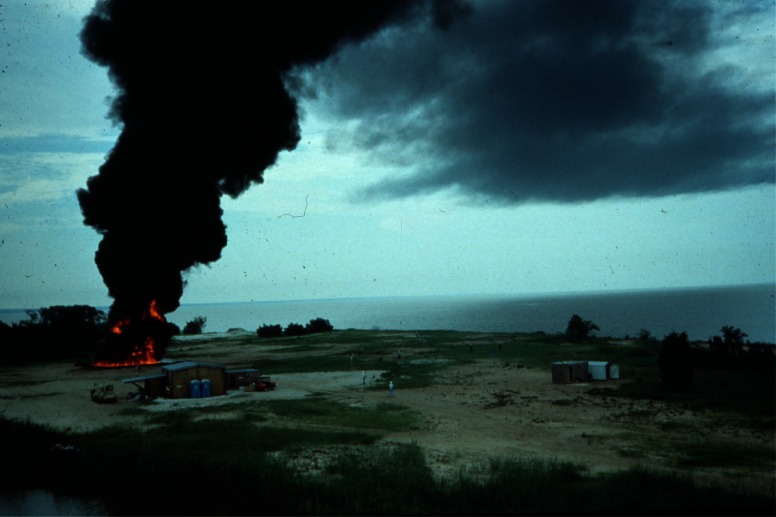
U.S. Coast Guard Safety and Fire Test Detachment mesoscale burn facility.

**Fig. 5:**
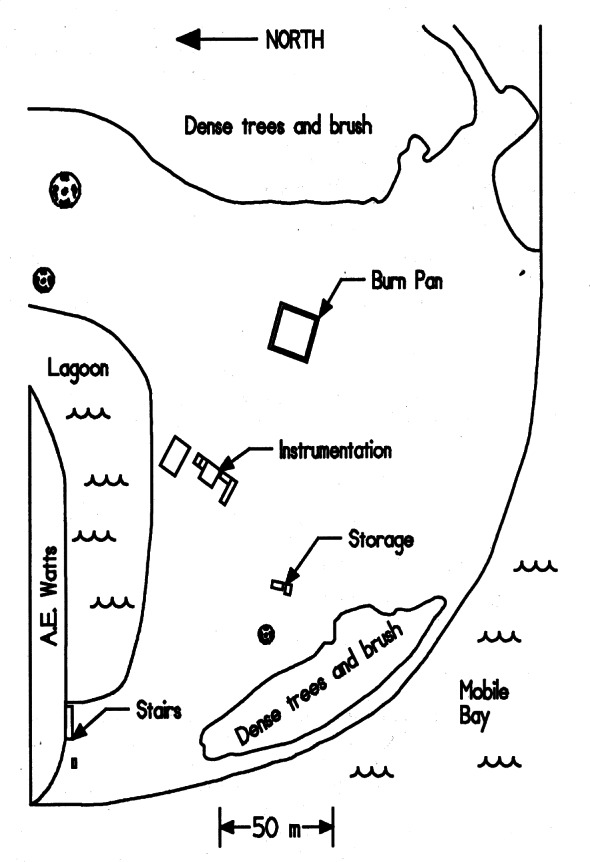
Site plan of U.S. Coast Guard mesoscale burn facility on Little Sand Island, Mobile Bay, Alabama.

The mesoscale burns of crude oil were carried out under the direction of NIST at the United States Coast Guard (USCG) Fire and Safety Test Detachment facility on Little Sand Island in Mobile Bay Alabama. Little Sand Island is approximately 0.2 km^2^ in size and includes decommissioned ships docked in a lagoon. The ships and facilities on the island have been used for a wide variety of full-scale marine fire tests. Figure 4 is a photograph of a burn in progress, and Fig. 5 is a plan view of the portion of the island used for the oil spill burns.

Burns were conducted in a nominal 15 m square steel burn pan constructed specifically for oil spill burning. The burn pan was 0.61 m deep and was constructed with two perimeter walls approximately 1.2 m apart forming an inner and outer area of the pan. The inside dimensions of the inner area of the pan were 15.2 m by 15.2 m. The two perimeter walls were connected with baffles and the space between the walls, which formed the outer area of the pan, was filled with bay water during the burns. The base of the pan was 6 mm thick steel plate and the walls were 5 mm thick steel plate. The tops of the walls were reinforced with steel angle to prevent warping during the burns. The base of the pan was located on ground level and was reinforced with steel beams on steel footers under the pan. Water fill pipes were connected to both the inner and outer areas of the pan. Water was pumped directly from Mobile Bay into both the inner and outer areas of the pan. The inner area of the pan was filled with approximately 0.5 m of water and the crude oil was added on top of the water. An oil spill containment dike approximately 0.5 m high was constructed 4 m from the outer edge of the pan.

Three different primary burn areas were used. These areas consisted of the full inner pan with an area of 231 m^2^ and partial pan areas of 114 m^2^ and 37.2 m^2^. The partial pan areas were achieved by partitioning a corner of the inner pan with 0.14 m by 0.14 m timbers covered with sheet steel. Plywood skirts 0.3 m deep were attached to the timbers below the water surface to prevent the oil from flowing under the timbers. An effective diameter was calculated for each of the rectangular burn areas. The effective diameter is the diameter of circle with the same area as the rectangular burn area used. The effective diameters for the three areas are 17.2 m, 12 m, and 6.88 m. Additional details of the construction, installed instrumentation, and operation of this major test apparatus for oil burns are given by Walton et al. [11].

Various means were explored to obtain samples from the wind blown smoke plume. Experience with smoke sampling using battery powered lightweight instruments in large laboratory tests indicated that the duration of sampling would have to be nominally 10 min. Instrument platforms evaluated for this purpose included towers, manned aircraft, fixed winged and helicopter remotely piloted aircraft, and balloons or mini-blimps [12]. It was decided that a tethered mini-blimp would be the primary means of positioning instrumentation for soot collection in the smoke plume. A 5.6 m long and 2.3 m diameter tethered helium filled mini-blimp could be controlled by ground personnel. Control and instrument payload tests using 15 m diameter fires were carried out at the Navy’s Farrier Fire Training Facility in Norfolk, Virginia to evaluate operational limits of the mini-blimp. This size mini-blimp can carry a 4 kg instrument package to one hundred meters above ground level. It can be readily moved from one location to another. Launch and recovery procedures are simple, and little time is needed to learn how to safely maneuver the blimp. Mini-blimps were used both in the mid-scale pan burn tests and in the at sea tests for positioning airborne instrumentation packages. Tethered mini-blimps were also used to position airborne weather stations about 50 m above the test site to continuously measure atmospheric conditions during experiments and transmit that data via radio to a ground station.

**Fig. 6:**
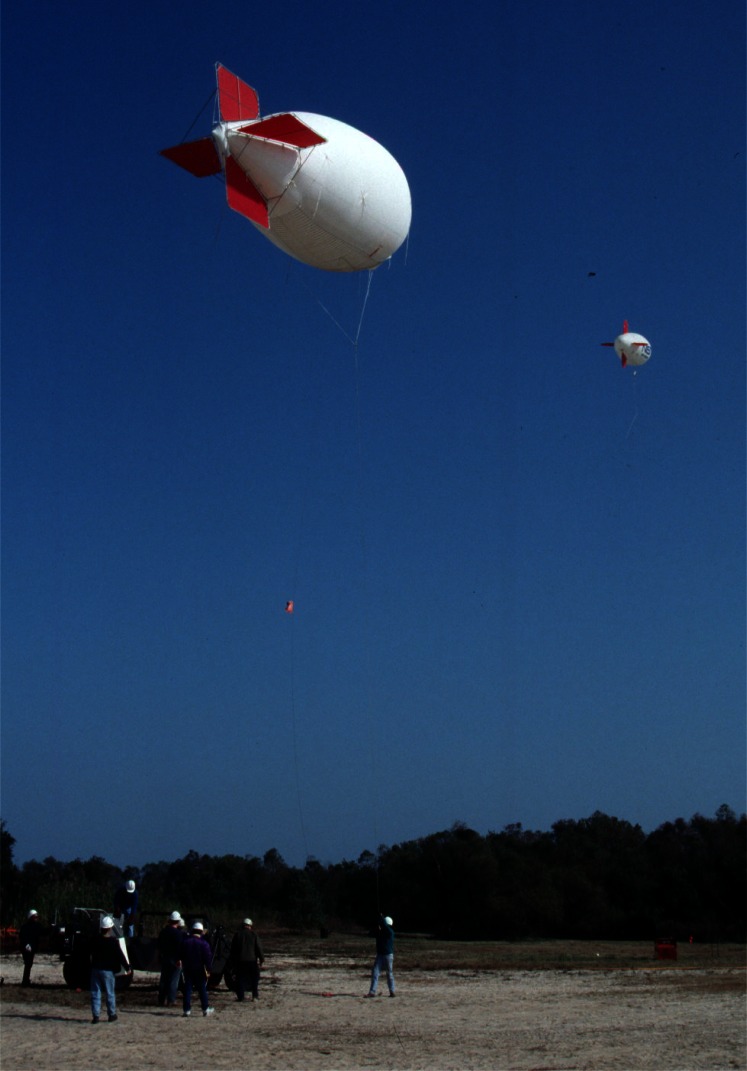
Mini-blimp used to support instrument packages for smoke plume gas and particulate sampling.

During burns at the Mobile, Alabama test site, airborne samples were collected for both laboratory analysis and analysis on the ground immediately following the burns. All sampling packages were suspended approximately 60 m below the mini-blimp, Fig. 6. The mini-blimp was positioned downwind from the fire with the sampling package centered in the smoke plume. The elevation and downwind position of the sampling package varied with each burn as a function of the plume position. Typically, sampling packages remained in the plume for 600 seconds. That permitted an adequate sample to be collected and allowed the natural fluctuations in the plume to be averaged. Since the lift capacity of the mini-blimp was limited, in general only a single sampling package could be deployed at a time. In some cases, where the burn was of sufficient duration, two packages were deployed sequentially.

The sampling packages consisted of battery-powered pumps that drew samples through filters and discharged a portion of the gas into a collection bag. Filter samples were analyzed in the laboratory for PAH and VOC concentrations. Particulate size distribution was measured using a cascade impactor. In addition, smoke particulate was collected on a thermophoretic transmission electron microscope grid (TEM grid) and analyzed using a transmission electron microscope to determine particle shape.

### 3.4 Offshore Experiments

**Fig. 7:**
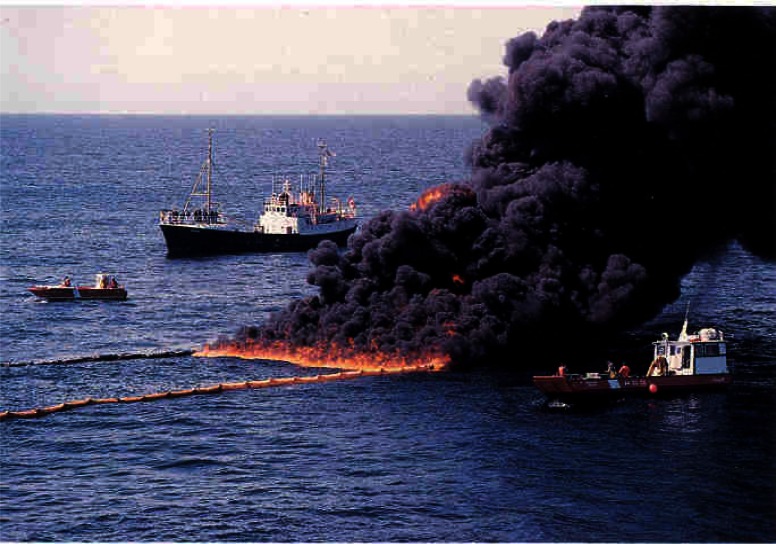
Oil burns in NOBE experiment.

**Fig. 8:**
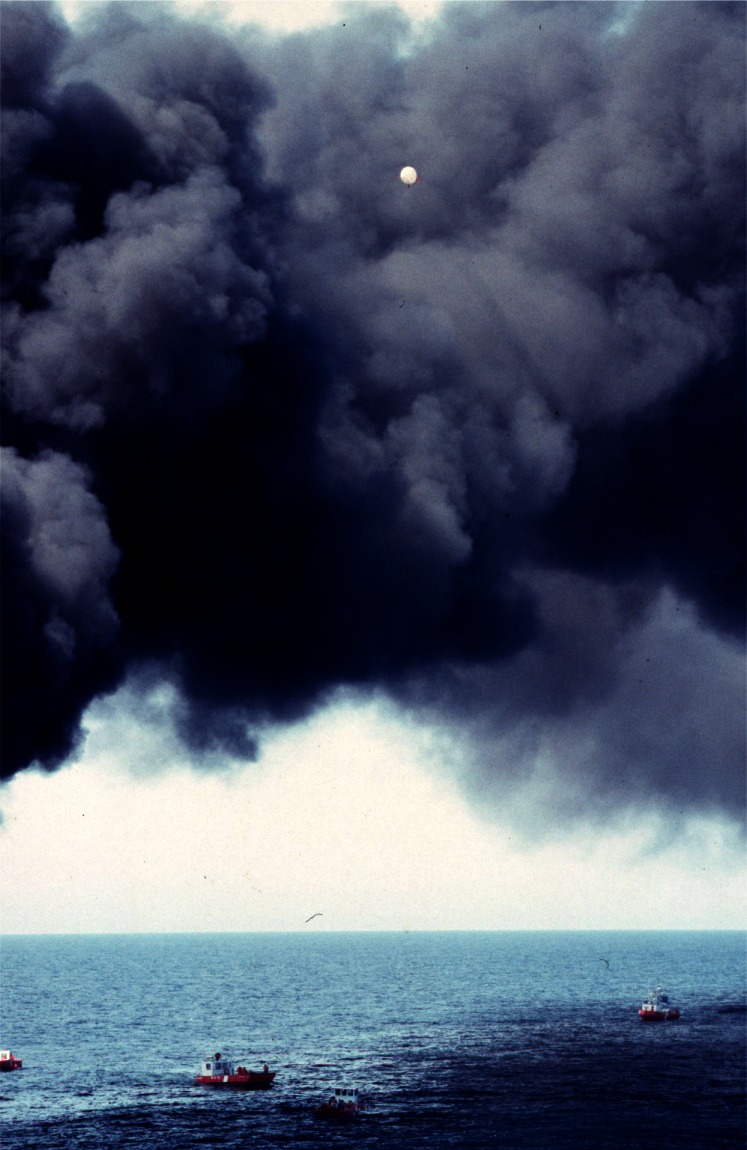
NIST samples smoke plume approximate 300 m downwind of the burning oil using instruments suspended beneath the mini-blimp (top-center of photograph above smoke plume) tethered to support vessel (lower-left).

Everything that was learned about measuring smoke properties in plumes from large fires on land was utilized in a large scale burn at sea burn experiment conducted under the direction of Environment Canada 25 km off the coast of Newfoundland, Canada near St. John’s on August 12, 1993. This experiment known as NOBE (Newfoundland Offshore Burn Experiment) included the sponsorship and participation by more than 25 Canadian and U.S. government agencies and industries. This experiment provided the opportunity to make measurements and evaluate equipment performance at a burn at the anticipated scale of actual response operations at sea, Fig. 7. NIST took on the responsibility for particulate and gas sampling from the smoke plume. Instrument packages developed by NIST were suspended below a helium filled mini-blimp tethered to a vessel stationed approximately 300 m down wind of the burning fuel contained in a fire resistant boom. The elevation of the mini-blimp and its position could be adjusted to keep the instrument package positioned in the plume for sample collection, Fig. 8.

## 4. Experimental Results

### 4.1 Burning Rate

The burning rate of oil on water was measured to quantify the removal rate potential of in situ burning. The burning rate per unit area of fuel spills is known to initially increase with increasing pool diameter, but reaches a plateau for large fires. Large-scale experiments were conducted using two different methods to determine the fuel mass loss rate during burning—measurement of initial volume and burn time and measurement of fluid pressure changes. For application to actual response, only the large scale experiments (burn diameters greater than 5 m) are of interest. These measurements were conducted at the Mobile, Alabama facility, over a period of several years. Generally the measurements from the last series of test are the most reliable for burning rate determinations as significant advances were made in measurement technique based on experience.

The burning of the crude oil was observed to take place in four distinct phases. The four phases were; 1) spreading, 2) steady burning, 3) steady burning with boiling of the water below the oil layer, and 4) transition to extinction. The spreading phase lasted from 80 s to 180 s as flames spread over the surface from the single ignition point on the upwind side of the pan to cover the entire fuel surface. Once the entire oil surface was covered with flames, the burning continued at a steady rate until the water below the oil surface began to boil. The onset of boiling was characterized by a noticeable increase in fire generated sound which resembles sizzling and bubbles breaking through the oil surface. During boiling the burning rate increased to a steady rate which was greater than the rate prior to boiling. When the fuel was nearly consumed, the fire began a transition to extinction. This was characterized by areas of the oil surface with no visible flames. Frequently, there were oscillations in the burning behavior with increased and decreased burning area and transition to and from boiling. The burning area decreased toward the downwind side of the pan until extinction.

The burning rate or the rate at which the oil was consumed during burning was estimated in the most recent tests from the liquid level in the pan as measured by the pressure transducer [11]. The output of the pressure transducer was calibrated in salt water and converted to oil depth using the specific gravity of the oil. The specific gravity of the oil was 0.846 ± 0.001 as measured using the mechanical oscillator technique with an accuracy of ± 0.001. The salt content of the water in the pan was measured before each test using the sodium ion electrode method with an accuracy of ± 0.01 %. The oil surface regression rate was calculated using a least squares linear fit of the pressure transducer output over the time from full pan involvement to the beginning of extinction. The data showed no difference in the burning rate before and during boiling.

The specific mass burning rate (rate of mass loss per unit area) was calculated from the surface regression rate and the density of the oil. The heat release rate was determined by multiplying the mass loss rate by the effective heat of combustion for the crude oil (41.9 MJ/kg) [11].

**Fig. 9:**
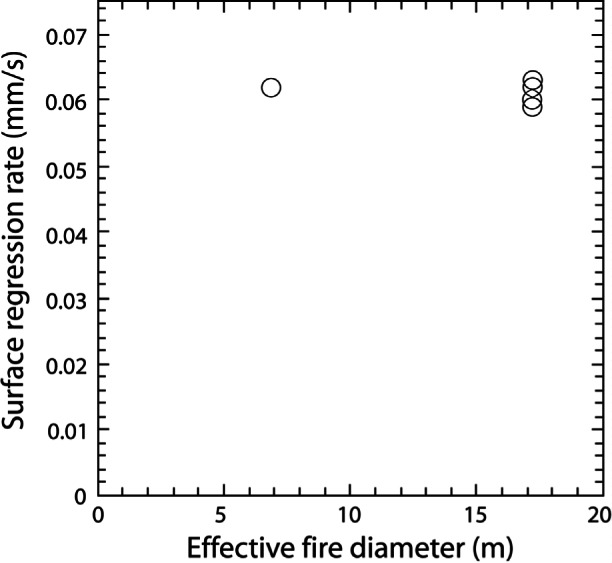
Crude oil surface regression rate for mesoscale burns.

Figure 9 is a graph of the surface regression rate as a function of the effective burn diameter. From this graph it appears that for the range of diameters used in the mesoscale burns there is no dependency of surface regression rate on burn area. The mean value is (0.062 ± 0.003) mm/s. The mean value for the burning rate per unit area is (0.052 ± 0.002) kg/s/m^2^ and for the heat release rate per unit area is (2180 ± 100) kW/m^2^. The scatter in the regression, burning and heat release rates was due in part to the variable nature of the burns. The wind direction and speed contributed to the wide variation in extinction behavior observed although it did not appear to affect the average burning rate.

### 4.2 Smoke

An important element of this study was the characterization of the smoke particulate, since it is the particulate that will ultimately lead to the health and environmental consequences. These impacts depend on the amount of smoke produced, the particulate size distribution, and the chemical makeup of the soot. NIST provided innovative measurement methods and new information on all of these topics. The Smoke Yield section describes the development and application of the Carbon Balance Method to determine the smoke yield from small and large oil pool fires. The results of the aerodynamic size distribution of the soot, which determines the deposition fraction in the respiratory tract, are reported from a range of crude oil types and fire sizes in the Size Distribution Section. The chemical makeup of the soot including the organic and graphitic components and the specific polycyclic aromatic hydrocarbons, some of which are known or suspected carcinogens, is described in the section on Polycyclic Aromatic Hydrocarbon Emission. To allow a more complete impact analysis, data is also included in this section on the PAH distribution of the original crude oil and of the burn residue. Two additional sections are included to provide a more detailed characterization of the soot agglomerates and their properties. The soot particles are agglomerates made up of nearly spherical primary particles. The size distribution of these spheres and their dependence on fire size is discussed in the Primary Sphere Section. The agglomerates grow as a result of particles colliding and sticking, and the effect of this process on the size distribution is reported in the Smoke Aging Section.

#### 4.2.1 Smoke Yield

Smoke yield is defined as the mass of smoke aerosol generated per mass of fuel consumed. The smoke aerosol collected during these experiments contained both solid material (graphitic carbon) and condensable hydrocarbons from the fire plume. Two methods for determining smoke yield were used in this study. The first was the flux method which measured the smoke collected on a filter and the mass loss from the burning specimen [13, 14]. This type of measurement worked well in a laboratory test environment where all the products of combustion were collected and drawn through an exhaust stack. The defining equation for smoke yield based on the flux method *ε*_1_ is given by:

$$\varepsilon_{1}=\left(\frac{m_{s}}{m_{f}}\right)\phi ,$$

where *m*_s_ is the smoke mass collected on the filter from an exhaust stack sample, *m*_f_ is the fuel mass consumed during filter collection, and *ϕ* is the ratio of mass flow of air through the stack to the mass flow through the sample filter.

The second method of determining the smoke yield is referred to the carbon balance method [14, 15]. This method required a determination of the ratio of the smoke mass in a given volume to the total mass of carbon in the form of gas or particulate in the same volume. This was accomplished by dividing the smoke mass collected on a filter to the sum of the smoke mass and the mass of carbon contained in the forms of CO and CO_2_. The equation for calculating smoke yield *ε*_2_ as expressed in terms of CO_2_ and CO concentrations is given by:

$$\varepsilon_{2}=\frac{f\;m_{s}}{\left[m_{s}+0.012n_{t}(\Delta \phi \left(\text{CO}\right)+\Delta \varphi \left({\text{CO}}_{2}\right)\right]}.$$

The quantity *f* is the carbon mass fraction of the fuel (0.855 for the crude oil blend used in this study), *m*_s_ is the mass of the smoke sample collected on a filter, *n*_t_ is the number of moles of air sampled, and the constant 0.012 represents the molar mass of carbon in kilograms. The quantities Δ*φ* (CO) and Δ*φ* (CO_2_) are the volume fractions of CO and CO_2_ of the gas sample taken during the test minus the ambient background concentrations of these gases. In this equation, the other carbon containing gases were neglected based on observations from laboratory scale open burns that these other species made up 2 % or less of the total carbon emitted by the flame [14]. One other approximation was that the smoke collected is pure carbon. In fact, the smoke is mainly “graphitic” carbon with an estimated carbon content by weight of 95 % or greater. This, together with the fact that *m*_s_ is small relative to the other terms in the denominator of the equation for *ε*_2_, less than 20 % of the total, leads to at most a 1 % uncertainty in the value of the smoke yield for this approximation.

##### 4.2.1.1 Smoke Yield Measurement

**Fig. 10:**
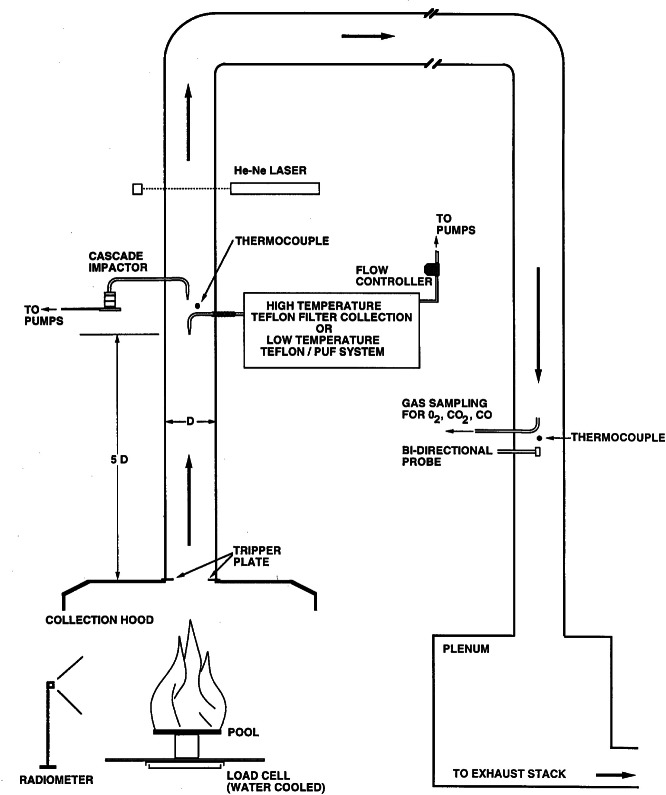
Schematic diagram of the crude oil pool fire burning apparatus.

These two methods of measuring smoke yield were used in the NIST Large Calorimeter. The experimental facility accommodated oil pool fires up to 0.6 m in diameter. A schematic drawing of the instrumentation is shown in Fig. 10. A photograph of a crude oil fire in this hood was shown previously in Fig. 2. Liquid pool fires were situated under the 2.4 m × 2.4 m collection hood with an adjustable exhaust rate up to about 2 m^3^/s (4000 ft^3^/min). A tripper plate at the stack inlet assured uniform mixing of combustion products and dilution air before the gases were sampled five duct diameters downstream of the inlet. The mass loss of the burning fuel floated on a deep water layer was monitored using a water cooled load cell with a sensitivity of about 3 grams. Thermal radiation from the fire was measured with a Gardon-type radiometer located several pool diameters from the pan.

Energy release rate from a fire was determined using oxygen consumption calorimetry. This method is based on the fact that the heat of the combustion is about 13 kJ/g of oxygen consumed for all hydrocarbon fuels [16]. Thus, measurements of O_2_ concentration and total flow rate through the stack made downstream of the horseshoe section in the duct were used to calculate the O_2_ consumption rate and proportional energy release rate of the fire.

**Fig. 11:**
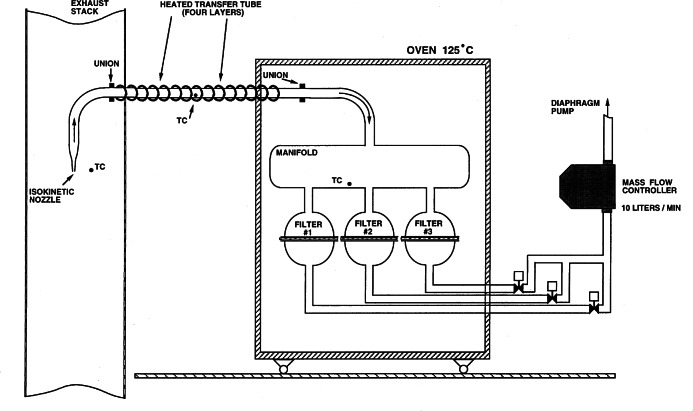
High-temperature Teflon filter collection apparatus. TC indicates a thermocouple.

The filter collection system, illustrated in Fig. 11, allowed for the sequential collection of three filter samples over the course of a pool fire test. The transfer line, manifold, and filter holders were all heated to match the stack temperature during the burn. This was done to minimize the evaporation/condensation of the smoke aerosol during transport or on the filter and to minimize the thermophoretic driven deposition of particles on the walls. The sample flow, about 10 L/min, and nozzle inlet, 4.8 mm, were selected to insure isokinetic sampling. The all glass construction of the filter collection system allowed ready inspection and ease of cleaning. The transmittance of a He-Ne laser, λ = 633 nm, through the smoke in the stack, 0.48 m path length, was monitored with a photometer and ratioed to the incident laser intensity to compensate for variations in the source intensity. The design of the photometer is reported by Babrauskas [17].

The gas sampling probe, pitot tube, and thermocouples were located downstream of the horseshoe section of duct. The gas velocity in the stack was determined from the pitot tube measurement together with the thermocouple reading. The sampled gas was cooled with a dry ice trap to remove water vapor, filtered, and then drawn into the gas analyzers. Non-dispersive infrared detectors are used for monitoring the concentrations of CO and CO_2_. This information was used in determining the smoke emission based on the carbon balance method.

##### 4.2.1.2 NIST Large Calorimeter Results

A summary of the results from four pool burns is given in Table 1. Increasing the pool size from 40 cm to 60 cm more than doubled the heat release rate and the mass loss rate of fuel, but it had a relatively minor effect on the smoke yield and in the smoke properties. The emission of smoke was high with *ε* about 0.10 (10 % conversion to smoke) and the mass specific extinction coefficient, *K*_S_ = 9.1 m^2^/g, was similar to what has been observed for other soot producing fuels. Over 60 % of the smoke aerosol was in the submicrometer aerodynamic size range, which is indicative of a long atmosphere residence time and potentially high deposition in the lower respiratory tract. More details on the size distribution and optical property measurements are discussed below.

##### 4.2.1.3 Field Measurement Equipment

**Fig. 12:**
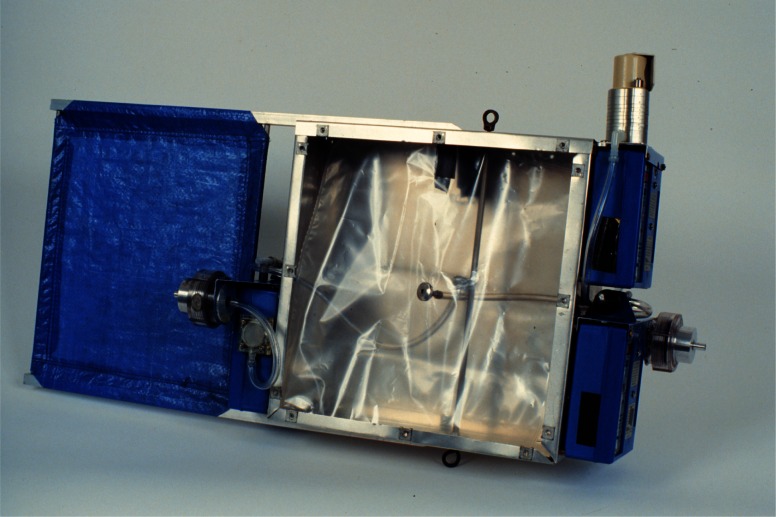
Assembled smoke sampling package.

**Fig. 13:**
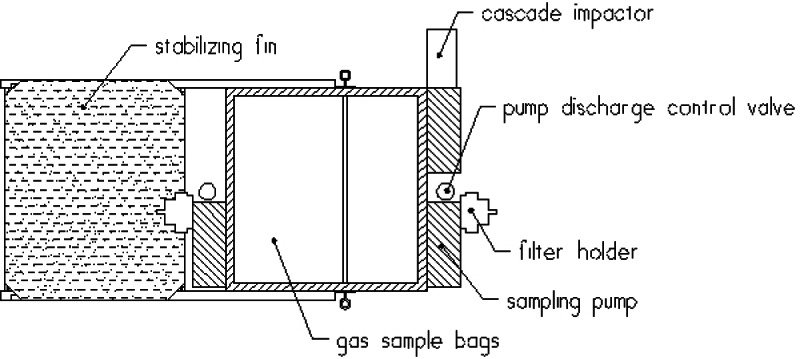
Diagram of smoke sampling package components.

The development of small, light-weight, battery-powered pumps used for personal environmental air sampling made it possible to assemble a smoke yield measurements system that was light enough to be flown by a 12 m^3^ mini-blimp. The Airborne Smoke Sampling package (ASSP) shown in Fig. 12 and Fig. 13 consisted of two 47 mm diameter aluminum filter holders, a mini-cascade impactor, three constant flow pumps (Gillian^1^ Model HFS 513A), and a light-weight aluminum enclosure for holding two 5 L polyvinyl fluoride (PVC) plastic sample bags. A similar approach to sampling, including the use of a constant flow pump and gas sampling bag was used by Ward [18] for sampling biomass fires from a tower. A more detailed description of the development of a prototype design of the ASSP is reported by Lawson et al. [19].

##### 4.2.1.4 Laboratory Test of Field Equipment

Initial testing of the ASSPs involved simultaneous measurements with the ASSP and the calibrated flux method smoke yield system described above. The facility at NIST collected all smoke and gases from fires, up to about 400 kW, in a hood, with a known fraction of the total flow passing through a filter. During the filter collection process, the fuel’s mass loss was measured. The testing involved fuel oil pool fires with a 40 cm diameter pan. A layer of oil 1 cm thick was floated on water. The burning rate of the fuel increased for about 60 s, was steady for about 400 s, and, just before flame extinction, increased greatly for 10 s to 20 s during boilover. The heat release rate from the fires was about 100 kW. The smoke collection was started in each case 1 min after ignition and continued for 5 min during the steady burning phase. It is important to collect over the same time period, since the smoke yield increased on the order of 10 % to 20 % with time during the steady burning phase [13].

The ASSP probe was located in the 50 cm diameter exhaust stack at the same height as the fixed laboratory system and within 5 cm laterally. Both sampling systems were operated with flows and probe opening diameters that would provide equivalent face velocities, and both systems sampled for the same time period. As can be seen from the results in Table 2, the field system’s average value was within 13 % of the average of results obtained by the laboratory flux method.

##### 4.2.1.5 Intermediate Scale Oil Burn Studies in Japan

A joint US-Japan study was carried out to extend the data base for quantitative smoke yield and primary particle size for the burning of crude oil within an enclosure to include a 1 m pan diameter and a 2.7 m × 2.7 m pan [20]. The experiments were performed at the large scale NRIFD in Mitaka, Tokyo, Japan illustrated in Fig. 3. To ensure reliable results in the present study triplicate tests were performed at both scales and two measurement approaches were employed. A key feature of the study was the use of the carbon balance method for the quantitative measurement of smoke yield.

**Fig. 14:**
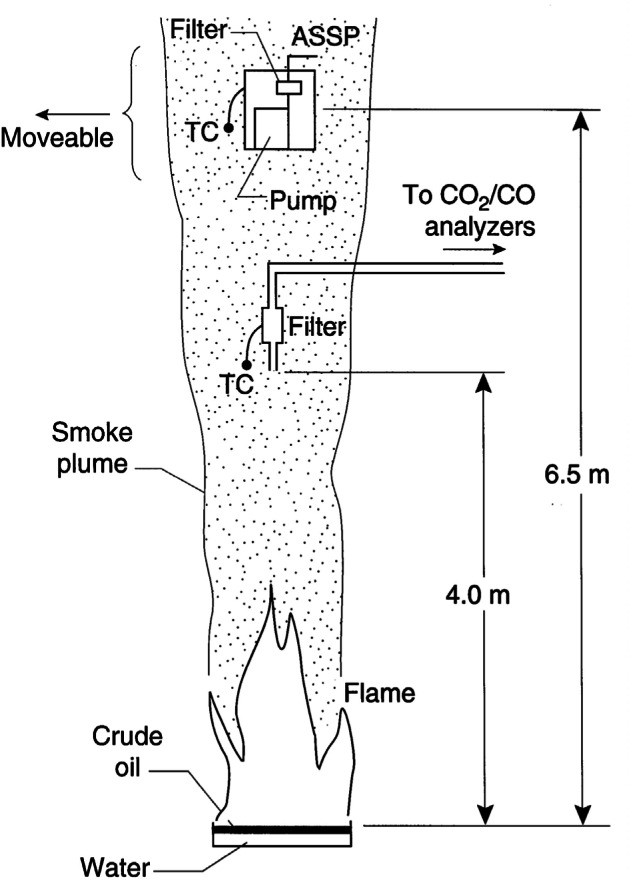
Schematic diagram of the location of the smoke sampling equipment relative to the location of a 1 m crude oil pool fire. The gas sampling bag from the airborne smoke sampling package (ASSP) is not shown.

Figure 14 shows a schematic diagram of the experimental setup. A mixture of 80 % Murban and 20 % arabian crude oil was burned in pans placed at the center of the test facility, which has an open area 24 m × 24 m under a 20 m high ceiling. The crude oil was burned in a 1 m diameter circular pan and in a 2.7 m square pan with an oil layer of 2 cm floating on water. This same facility was used to measure the burning rate and radiant output before and during boilover [21, 22].

Two different procedures, both based on the carbon balance method, were used for measuring the smoke yield. In one, a sampling probe was positioned 4 m above the pan for the 1 m fire and in the exhaust duct of the facility for the case of the 2.7 m square pan. The smoke/gas entered a 6.5 mm diameter sampling probe at near isokinetic velocity of about 5 m/s for the smaller pan and about 10 m/s for the larger pan. The smoke particulate was collected on a ceramic filter while the gases flowed to a CO/CO_2_ nondispersive infrared analyzer. The nominal average values for volume fraction were 2.0 × 10^−3^ for CO_2_, 4.0 × 10^−5^ for CO, and 90 °C for both the 1 m and 2.7 m pan sizes. For the 1 m pan diameter, near the end of the typically 10 min burn, boilover occurred resulting in enhanced burning and an increase in temperature by about 150 °C. The second method utilized the airborne smoke sampling package (ASSP) described above. One of the objectives of these tests was to validate the ASSP for determining smoke yield by sampling smoke particulate and combustion gases produced by a large buoyant plume.

The average smoke yields obtained by the two methods for the 2.7 m square pan agreed well, 0.148 ± 0.012 (three tests for ASSP) vs 0.149 ± 0.015 (three tests for the continuous sampling). The average yield for the 1 m pan was 0.100 ± 0.008 (four tests for the ASSP) vs 0.061 (two tests for continuous sampling). The uncertainty range is the standard deviation for the repeat tests. One reason for the lower value for the continuous sampling was that the smoke is collected throughout the burn including the boilover period during which the yield was reduced [23]. The smoke was not collected during boilover by the ASSP to avoid damage to the plastic components (collection bag, plumbing, and pump housing). A difference in yield for the two approaches was not expected for the larger pan because the boilover effect was minimal. The key observation was that the smoke yield increased by about 50 % as the pan size was increased from 1 m to 2.7 m. The corresponding burning rates for the two pan sizes was approximately 0.022 kg/s and 0.26 kg/s.

##### 4.2.1.6 Scale Dependence of Smoke Yield

**Fig. 15:**
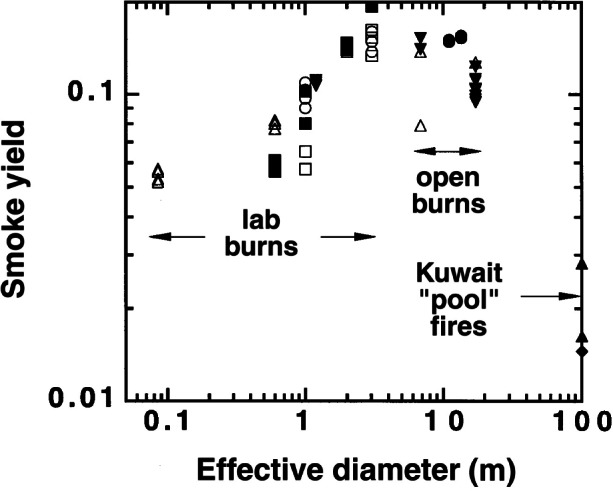
The effect of pan diameter on the smoke yield of burning crude oil including data from Ref. [11] ▼, Ref. [20] □, ○, Ref. [21] ■, Ref. [24] ●, Ref. [25] △, Ref. [26] ▲, and Ref. [27] ♦.

In Fig. 15 the smoke yield is plotted versus pool diameter. We defined the effective diameter of the 2.7 m square pan as the diameter of a circle (3.05 m) with area equal to the square pan. Fig. 6a includes other crude oil fires with “pan sizes” ranging from 0.085 m to 100 m [11, 20, 21, 24, 25, 26, 27]. The present study with pan sizes of 1 m and 3.05 m matched two of the sizes used in a previous study [21]. The average yields obtained by the ASSP in the present study were 0.148 and 0.100 compared to 0.194 (1 test) and 0.087 (3 tests) obtained in the previous study [21] for the 3.05 m and 1 m pan size, respectively. Our present experiments confirmed the trend of increasing smoke yield with increasing pan size though the magnitude of the increase, about 50 %, was less than the more limited results of the previous study [21].

The data from 2 m to 15 m based on five studies [11, 20, 21, 24, 25] with five types of crude oils (Murban, Arabian light, Louisiana crude, Murban-Arabian light mixture, and Newfoundland crude) appear to be independent of size; with one exception the data fall in the range 0.13 to 0.16. For the pan sizes larger than 3 m, the burns were performed outside where the ambient wind may affect the smoke yield.

The results from two series of tests at 17.2 m are significantly lower than the results from 2 m to 15 m. The results from one series [11] range from 0.101 to 0.111 with a mean of 0.107 while the other was a single test with a value of 0.127 [25]. The cause for an apparent decrease is not known.

#### 4.2.2 Smoke Particulate Size Distribution

The particle size distribution of smoke aerosols is important for the evaluation of smoke plume dispersion and health effects. Due to the irregular shape of smoke particles, which consist of agglomerates of small spherules, the particles are typically classified by any one of a number of different equivalent diameters. In this study, the aerodynamic properties of the smoke aerosol are of primary concern, necessitating the use of equivalent aerodynamic diameter. The equivalent aerodynamic diameter of an irregularly shaped particle is the diameter of a smooth spherical particle, having a unit density of 1 g/cm^3^, with the same terminal velocity as the smoke particle falling in air under the influence of gravity.

Particle size was measured during the steady portion of the fires using two particle impactors, each with eight stages and a backup filter. The impactors are built in such a way that a number of overlapping perforated disks, or stages, force the particles to change direction and follow the bulk flow of air through the holes in the stage. Some of the particles, however, are unable to navigate this path due to their inertia and are deposited on the stage. Each stage is designed to collect particles of a certain size range, specified by the manufacturer as the cutpoint diameter of the stage, which is the aerodynamic diameter that is collected at 50 % efficiency. The cutpoint diameter decreases with each successive stage of the impactor. The cutpoint sizes for the impactors in our study range from 0.4 µm to 20 µm.

Two different types of impactor were used in this experimental series. The first was a full size unit, operating at a flow rate of 28.4 L/min. Due to the large size of this impactor, the mass collected on each stage is greater, as is the quantity of particulate collected. This combination of features helped reduce the uncertainty in the particle size measurements.

The second impactor used in this study was primarily designed to be worn by workers to monitor particulate exposure levels in the workplace. Due to the small size of this impactor, it was also used as part of an airborne smoke plume sampling package for large scale fires. As a consequence of the size and weight advantages, however, the flow rate of air sampled by the impactor was only 2.0 L/min. The capacities of the stages are also small, limiting the quantities of particulate that could be collected.

The sizes of smoke particles produced by the burning of Louisiana, North Slope, and Cook Inlet crude oils were reported by McGrattan et al. [28]. The distributions were repeatable. The size distribution for all of the crude oils for 1.2 m pan diameter were similar with about 65 % of particulate mass with aerodynamic diameter less than 1 µm. In reference to health concerns, the plots indicated that approximately 80 % to 99 % of the smoke aerosols collected had aerodynamic diameters of 10 µm or less. This is significant since the quantity of particulate matter below 10 µm cut size was a parameter used by the United States Environmental Protection Agency to gauge particulate pollution in ambient air [29].

**Fig. 16:**
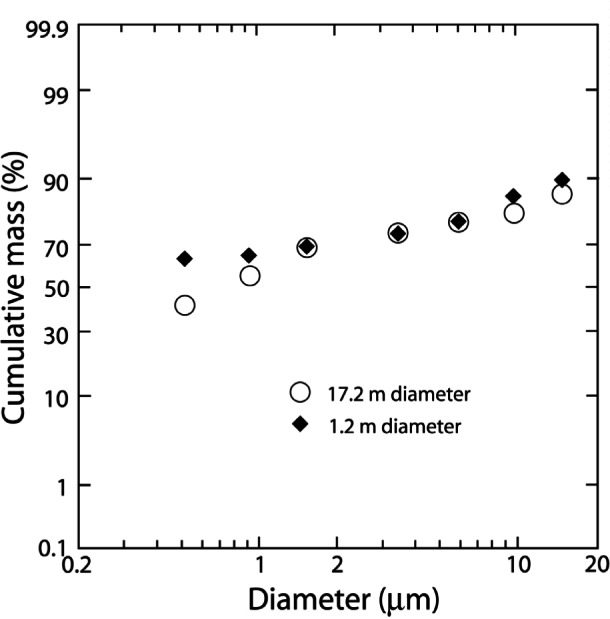
Smoke aerosol size distribution for Louisiana crude oil using the personal impactor.

**Fig. 17:**
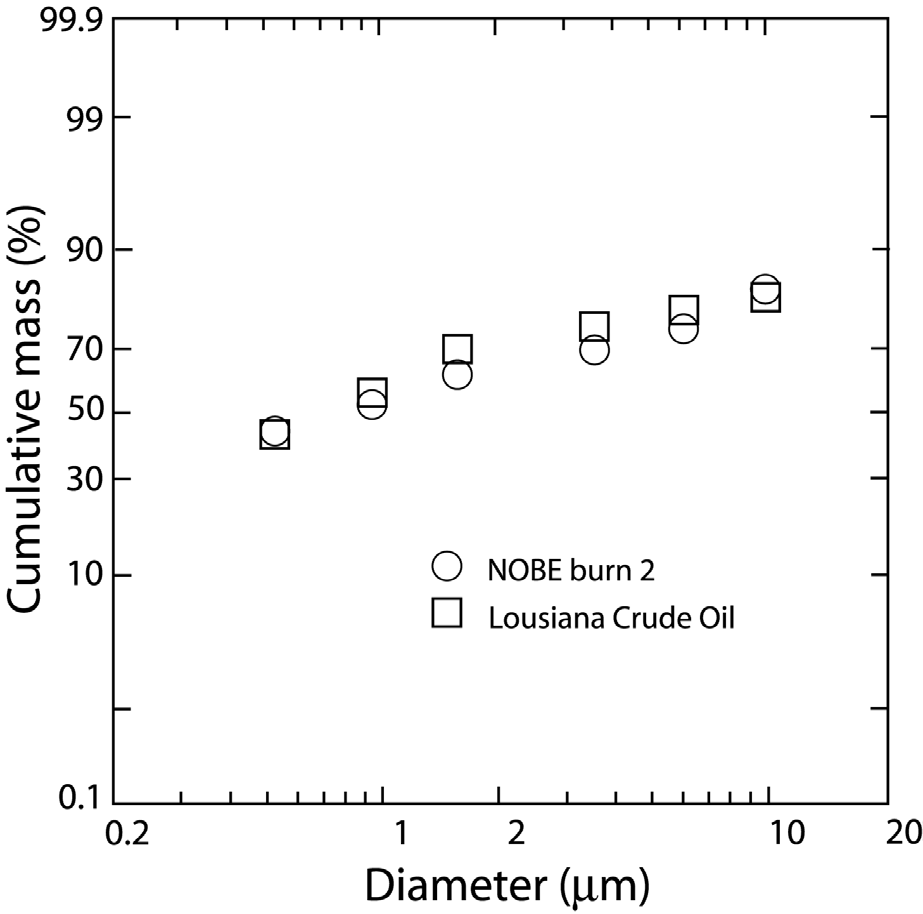
Comparison of aerodynamic size distribution for two 17.2 m diameter crude oil burns.

Smoke particle size measurement from all three of the oils showed nearly identical results, indicating that the trends observed for the Louisiana burns may be extrapolated to both Cook Inlet and North Slope oils. The particle distributions from the 1.2 m burns were also comparable to previous results for 17.2 m burns, illustrated by Fig. 16. Therefore, a similar distribution would be expected for a full scale burn of North Slope or Cook Inlet crude oils, resulting in 80 % to 99 % of the smoke aerosols meeting the under 10 µm criterion. Figure 17 shows the cumulative size distribution of smoke particulate for the 17.2 m effective diameter mesoscale Louisiana crude oil burn and the 10.1 diameter Newfoundland Offshore Oil Burn [30]. The two size distributions were similar and the cumulative mass of particulate below 10 µm was over 89 % for both tests. These distributions represented the smoke aerosol in the portion of the plume close to the fire, however, and would be expected to change as the plume progressed and the mechanisms of agglomeration and settling became significant. The effect of agglomeration on the size distribution is discussed below in the section entitled Effect of Aging on Smoke Properties.

##### 4.2.2.1 Primary Sphere Size

**Fig. 18:**
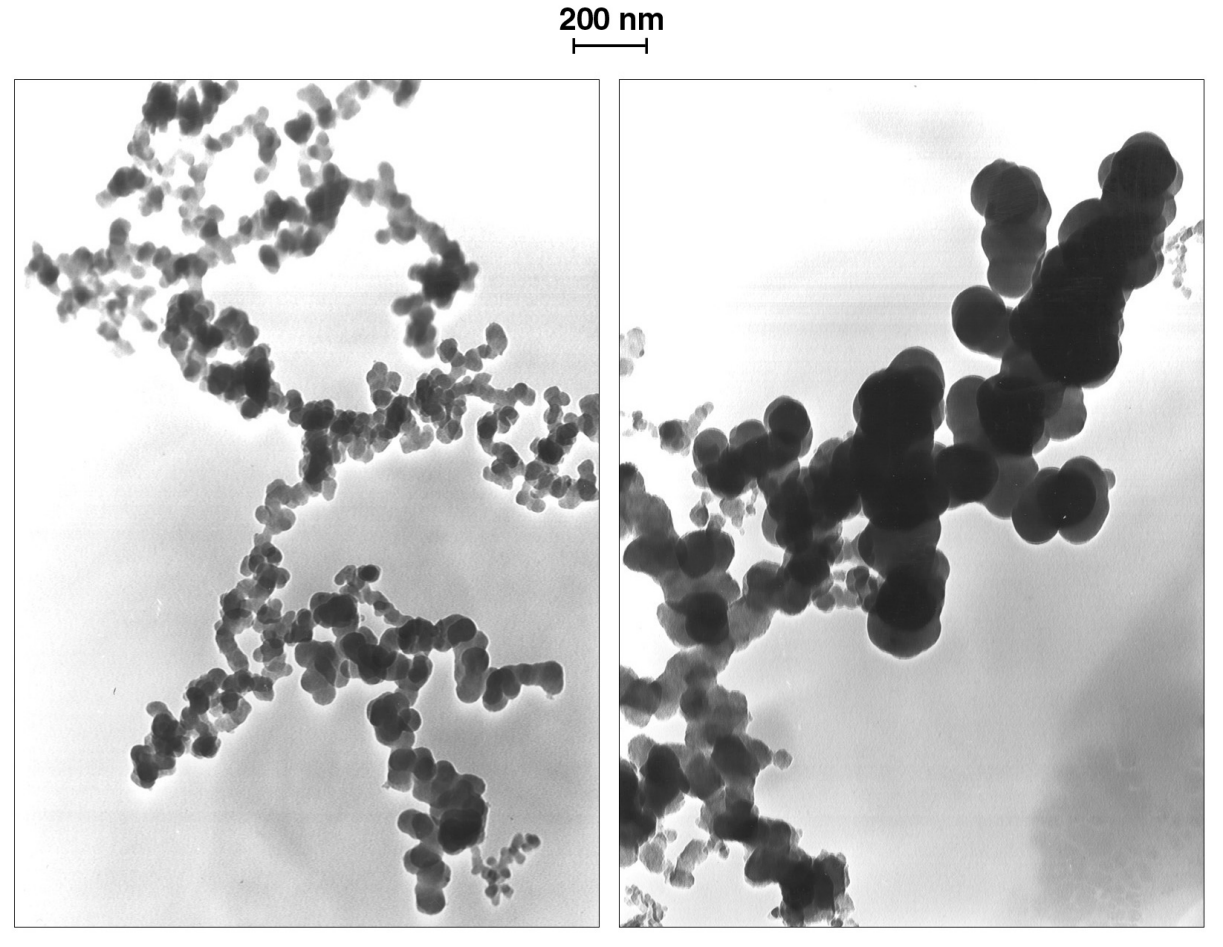
TEM photographs of smoke collected from crude oil fires for 1 m diameter pan (left) and 2.7 m × 2.7 m pan (right).

The soot consists of agglomerates made up of nearly spherical particles attached in a cluster. The primary sphere size is important in regard to the optical properties of the soot and the total surface area of the smoke agglomerates. A series of experiments were carried out at NRIFD to assess the effect of fire size on the primary sphere diameter [20]. Transmission electron micrograph (TEM) grids were attached to the ASSP using double stick tape. During a fire test, the smoke plume would flow past the TEM grids on the ASSP as indicated in Fig. 14, and soot agglomerates would deposit on the grids. The major deposition mechanism was thermophoresis resulting from the temperature gradient between the smoke and the TEM grid, which was cooled relative to the smoke plume by contact with the aluminum support structure of the ASSP. In Fig. 18 we show representative micrographs of the smoke collected from the 1 m and 2.7 m pans. The most striking feature is the apparent bimodal size distribution of large (100 nm to 150 nm) and smaller (30 nm to 70 nm) primary spheres for the larger pan. Furthermore, the larger spheres are grouped together and the smaller ones are also grouped together.

The particle size analysis for each fire size was based on about 20 TEM micrographs taken at randomly selected locations on a single grid. A total of 404 spheres were sized for the 1 m diameter pan and 483 for the 2.7 m × 2.7 m pan. The spheres selected for sizing for each photograph were determined from a transparent template with 100 randomly selected points. In the case where a point appeared in a region of overlapping primary spheres, the closest identifiable sphere was sized. This procedure was chosen over sizing every primary sphere to obtain a broader sampling selection and to avoid the ambiguity of regions where individual spheres were difficult to enumerate. The overlap was more prevalent for the larger spheres. Also, as explained below, this method provided a more accurate volume distribution than obtained by sizing every sphere in a photograph.

The TEM photographs were taken at a magnification of 30 000× and enlargement glossy prints (about 2.4×) were prepared for sizing. The primary spheres were measured manually from the prints to the nearest 0.1 mm using a measuring reticule with a magnification of 6×. The spheres were then binned with the first bin 0.25 mm to 0.75 mm, the second 0.75 mm to 1.25 mm, etc.

The procedure we used to randomly select the spheres was biased toward the choice of larger spheres. The probability of “hitting” a sphere of diameter *D* with coordinates chosen at random is proportional to the cross sectional area of the sphere. Therefore, the empirically determined distribution, *Y*(*D*), is related to the number distribution by the following expression:

$$Y\left(D\right)=CD^{2}n\left(D\right)$$

where *C* is a proportionality constant.

Our interest was in the volume distribution, *V*(*D*), of the primary spheres since the optical properties and the health impact are better correlated with the volume or mass distribution rather than the number distribution. The volume distribution is proportional to *D*^3^*n*(*D*). Therefore, multiplying *Y*(*D*) by *D* gives a result proportional to the volume distribution. This method gives a more accurate measurement of the volume distribution than counting every particle on a fewer number of micrographs (fixed no. of particles sized), since the volume distribution is more similar to *Y*(*D*) than to *n*(*D*) (1st power of *D* multiplicative factor vs 3rd power). This was especially true for the large fires where a relatively small number of large spheres contributed a large fraction of the volume distribution.

It is convenient to define a normalized volume distribution, *V*^1^(*D*), where the integration over particle diameter gives unity.

$$V^{1}\left(D_{i}\right)=\frac{D_{i}Y\left(D_{i}\right)\Delta D}{\sum D_{i}Y\left(D_{i}\right)\Delta D}.$$

**Fig. 19:**
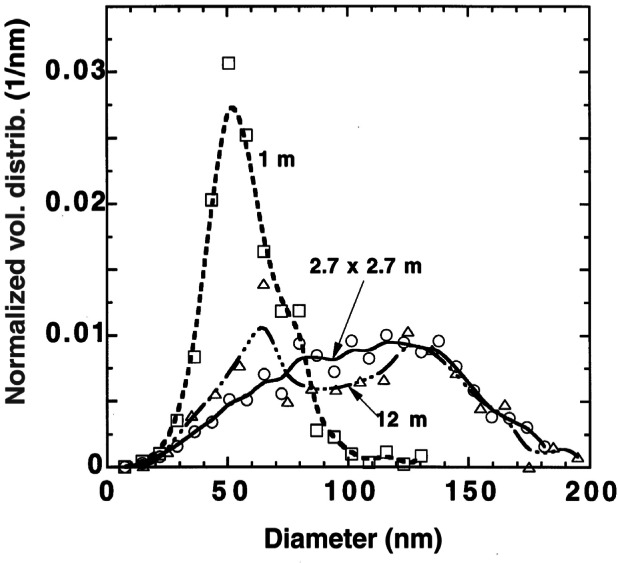
The normalized volume distribution of smoke from crude oil fires for a 1 m diameter pan, a 2.7 m × 2.7 m pan, and a 12 m diameter pan.

In Fig. 19 the volume distribution is plotted for the primary spheres for the 1 m pan diameter, the 2.7 m pan, and a 12 m diameter pan (317) points. A limited data set (83 spheres sized) for an 0.1 m diameter pan was similar to the 1 m pan result. In the case of the 12 m pan the fuel was Baton Rouge crude and the smoke was collected by the ASSP approximately 200 m from the flame-tip [25]. The bimodal character apparent in the micrographs from the larger fire was more apparent for the 12 m pan than for the 2.7 m pan. Apparently slight changes in mean sizes from sample to sample washed out the structure for the 2.7 m pan. From the volume distribution, the volume mean diameter, *D_v_*, was found to be 58 nm for the 1 m diameter pan, 106 nm for the 2.7 m × 2.7 m pan, 101 nm for the 12 m pan, and 51 nm for the 0.1 m pan.

Our observation that the volume mean diameter of the primary sphere increases by more than 80 % (58 nm vs 106 nm) as the pan diameter increases from 1 to 3.05 m appears to be new. This change was expected to affect both the optical and aerodynamic properties, since for a 106 nm sphere the optical size parameter, π*D*/λ = 0.7 for wavelength λ = 0.5 µm, and Knudsen number, 1.2, are both approaching the value 1, which marks a change from Rayleigh to Mie scattering and free molecular to continuum dynamics.

There are limited data for large scale fires. Our result for the volume mean diameter for a 12 m pool fire, 101 nm [25], is similar to the result for the 2.7 m × 2.7 m pan fire, 106 nm. Radke et al. [31] also observed large primary spheres for smoke collected from the burning of a 30 m diameter pool of aviation fuel. They comment that “most of the particles in the smokes consisted of two types of chain aggregates: one comprised of fairly uniform spheres with approximately 30 nm diameter and the others of spheres with approximately 150 nm.” Johnson et al. [32] report a primary sphere size of about 100 nm based on scanning electron microcopy of the smoke from the burning wells in Kuwait. Even if smaller diameter primary spheres in the range 30 nm to 60 nm were present, they would not be measurable by the scanning electron microscope.

Dobbins et al. [33] have studied the incipinet soot particles and found that individual microspheres composed of PAHs are formed low in a laminar diffusion flame. These microspheres grow by both a surface process as well by coagulation followed by coalescence. The microspheres low in the flame have a high content of PAHs. As they reach into the high temperature region of the flame they carbonize leading to particles with a much lower mole fraction of hydrogen, 0.15 high in the flame versus 0.36 low [33]. Once this happens the particles no longer coalesce upon contact but rather they form aggregates. This scenario suggests that the primary sphere size can be increased by decreasing the flame temperature or by increasing the residence time in the lower temperature region of the flame. The preceding suggestion is consistent with the well known result in the carbon black industry that a decreasing temperature produces a larger primary sphere size [34].

##### 4.2.2.2 The Effect of Aging on Smoke Properties

A facility illustrated in Fig. 2 was developed at NIST [35] to study how smoke aging would affect the properties of smoke. This is of interest because the size distribution of the smoke agglomerates may change as the plume moves downwind. The important mechanism of smoke aging analyzed with this facility was agglomeration as the clusters undergoing Brownian motion collide and stick together and sedimentation.

The fuel used in this study was Alberta Sweet crude oil with a boiling point range during distillation from 37 °C to 350 °C, density at 20 °C, 0.84 g cm^−3^, and flash point, 7 °C. The crude oil was burned in a 0.6 m diameter pan positioned under a 2.4 m × 2.4 m collection hood. A propane torch was used to ignite the 1 cm thick layer of oil floating on about 4 cm of water. A “tripper” orifice plate at the base of the exhaust stack ensured good mixing five diameters downstream where the smoke was drawn into the aging chamber.

**Fig. 20:**
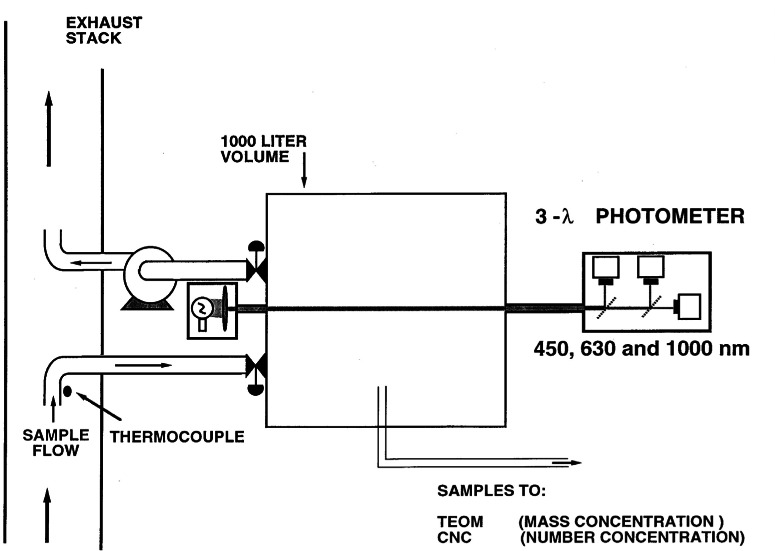
Schematic diagram of smoke aging apparatus with three wavelength photometer.

The chamber is a 1 m^3^ aluminum box which was lined with stainless steel to reduce corrosion from the hot combustion gases. Forty-eight mica resistance strip heaters were attached to the aluminum wall which evenly distribute the heat for wall temperatures up to 150 °C. In our study, the wall temperature was either at ambient conditions or near 100 °C, which was approximately equal to the exhaust duct temperature. An exhaust fan drew the smoke through the chamber at a flow of 0.13 m^3^/s. The chamber was connected to the exhaust stack via 10 cm diameter stainless steel tubing (Fig. 20). After the chamber is filled, two stainless steel butterfly valves, one on the inlet and one on the outlet, were simultaneously closed to capture a 1 m^3^ sample. Ports at opposite ends of the chamber allowed use of a three wavelength photometer for light extinction measurements.

The mass concentration of the smoke, *c*_m_, in the chamber was determined with a tapered element oscillating microbalance (TEOM). A continuous flow condensation nucleus counter (CNC) was used to monitor the number concentration, *c*_n_, during an aging experiment. Because of the high initial number concentration, the smoke was diluted about 50-fold before entering the nucleus counter. The initial mass concentration of the smoke was typically on the order of 100 mg/m^3^ and the concentration decreased by about a factor of two over a 2 h aging experiment as a result of particle sedimentation and wall loss. The filtered air introduced to balance the air being withdrawn for the TEOM and CNC was less than 5 % of the chamber volume and is not corrected for in these experiments.

The aerodynamic size distribution of the aging smoke [35] was measured with a cascade impactor similar to those described in the Sect. Size Distribution of Smoke. As the smoke aged, the mass median diameter increased from an initial 0.8 µm to 1.1 µm at 90 min. The corresponding settling velocities were 0.03 mm/s and 0.06 mm/s, respectively. Most of the change in the size distribution was for the particles sizes of 1 µm and less. For both the fresh and aged smoke, the size range below 8 µm corresponded to about 85 % of the total aerosol mass.

**Fig. 21:**
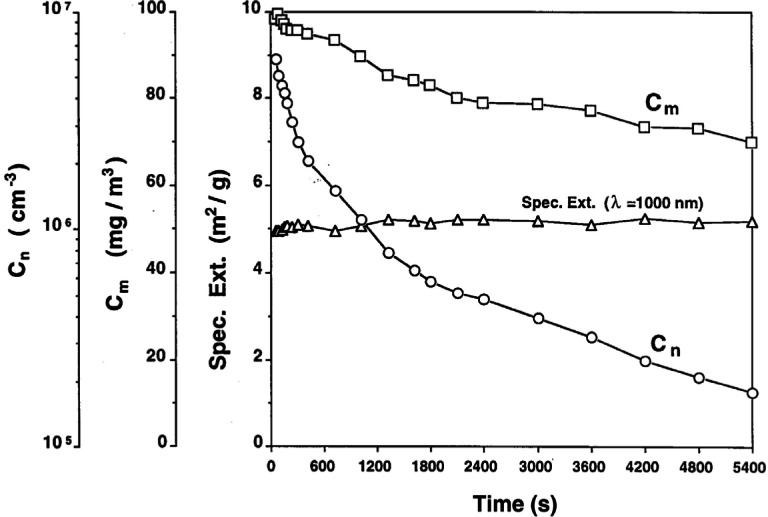
Soot mass concentration, number concentration and specific extinction vs time.

In Fig. 21. we present a graph of the measured mass *c*_m_ and number concentration *c*_n_, and σ_ext_ at λ = 1000 nm vs time for one test of 90 min duration. These results show that the mass concentration decreased in that time interval by 30 % owing to gravitational settling or diffusion to the chamber walls. On the other hand, the aggregate number concentration decreased by a factor of 24 caused mainly by cluster-cluster aggregation. From the decay of *c*_n_ with time a cluster-cluster aggregation rate of 15 × 10^−10^ cm^3^/s was calculated $\left(dc_{n}/dt=-\Gamma c_{n}^{2}\right)$. This compares with a value of 8 × 10^−10^ cm^3^/s found in similar experiments [35]. The specific extinction at λ = 1000 nm remained essentially constant despite the dramatic change in *c*_n_.

#### 4.2.3 Polycyclic Aromatic Hydrocarbon Content

Examining the levels of polycyclic aromatic hydrocarbons (PAHs) in the crude, residue, and smoke is critical for assessing the environmental impact, since some PAH species are believed to be carcinogenic [36 37 38 39]. It is known that there are some PAHs in the crude oil itself and also that PAHs are produced by the burning of hydrocarbon fuels, but there are no quantitative data on the relative amount of PAHs in the crude oil versus the amount emitted from burning the oil. In a study [23] involving the burning of Alberta Sweet crude oil in 2 mm, 3 mm, 5 mm, l0 mm, and 30 mm layers on water, 18 individual PAH components in the crude oil, in the burn residue, and in the smoke were identified and quantified. Two laboratories analyzed the particulate-and vapor-phase emissions as well as crude oil and burn residue for the PAH species. From the results of the fraction of oil burned, the amount of smoke generated per mass of fuel burned and the PAH content of the smoke, crude, and residue, an estimate of total PAHs released was obtained.

##### 4.2.3.1 PAH Measurement

Particulate- and vapor-phase samples were collected during burns of Alberta Sweet crude oil (boiling point range during distillation from 37 °C to over 350 °C, density at 20 °C, 840 kg/m^3^, flash point, 7 °C). The crude oil was burned in a 0.6 m diameter pan positioned under a 2.4 m × 2.4 m collection hood (Fig. 2 and Fig. 10). A propane torch was used to ignite the oil, which was floating on water that was approximately 4 cm deep. A water-cooled load cell, located under the pan, continuously monitored the mass loss rate during each burn. In the first phase of this study (phase I), the smoke produced by burning a 30 mm crude oil layer was collected at high temperature (100 °C) and at ambient temperatures (25 °C). In the second phase (phase II), crude oil layers of 2 mm, 3 mm, 5 mm, and l0 mm thicknesses were burned while particulate- and vapor-phase samples were simultaneously collected at ambient temperatures.

Two different filter systems located above the collection hood were used to collect the samples. The filter collection system used in phase I (Fig. 11) allowed for collection of up to three particulate-phase samples during each burn. The sample flow rate (10 L/min) and nozzle inlet diameter (0.44 cm) were selected for isokinetic sampling at an inlet velocity of approximately 11 m/s. For high-temperature collection, the transfer line, manifold, and filterholders were all heated to match the stack temperatures in order to minimize evaporation/condensation effects and losses to the walls of the collection system. For the low-temperature collection, a dilution section was inserted just downstream of the sampling tip and the heaters were switched off. Smoke was drawn through the isokinetic sampling probe into the dilution section where it was diluted 2:1 (mass basis) with 0 °C air, which cooled the smoke to within 2 °C of ambient. At both temperatures, a pair of Teflon filter samples were collected sequentially during the steady phase of the burn. After collection, each Teflon filter sample was weighed, sealed in Petri dishes, and stored under dry ice until transferred to a freezer (−20 °C).

**Fig. 22:**
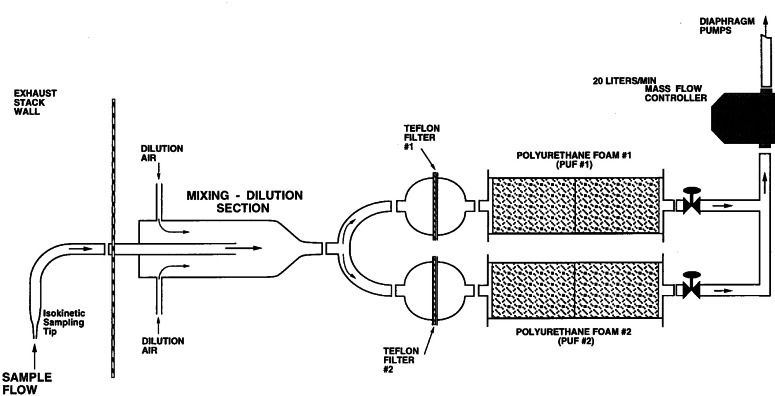
Low-temperature Teflon/PUF collection apparatus.

While higher collection temperatures were utilized to minimize the losses in the phase I experiments, lower collection temperatures were used in phase II to simulate the cooling that smoke experiences upon dilution in the atmosphere. The collection system used in phase II (Fig. 22) allowed collection of both vapor- and particulate-phase samples. With the same isokinetic sampling tip and dilution system from the earlier burns, the diluted smoke was collected in parallel by two sampling sets each consisting of a 64 mm diameter Teflon filter followed by two polyurethane foam (PUF) plugs in a glass tube, 37 mm diameter and 150 mm length. The PUF plugs were positioned downstream of the Teflon filters and collected both vapor-phase PAHs as well as those PAHs desorbed from the filter during the sampling process. In contrast to the phase I crude oil burns, during which samples were collected for short periods during the steady-state burning, in the phase II burns parallel sets of particulate-and vapor-phase samples were collected continuously from ignition until burning ceased. In order to collect sufficient mass for analysis, multiple burns were necessary for the 2 mm, 3 mm, and 5 mm oil layer thicknesses. Upon completion of a burn or series of burns, the Teflon filters were weighed and sealed in Petri dishes, while the PUF tubes were stored in aluminum foil. Both Teflon filters and PUF filters were stored under dry ice until transferred to a freezer. In addition to the Teflon and PUF filters, five samples of the crude oil before the burn and seven residue samples after the burn were also collected for chemical analysis. To determine the mass of oil remaining after the pool fire stopped burning, oleophilic batting was weighed and then used to soak up the unburned oil. Since the oleophilic batting preferentially adsorbed oil, only minute quantities of water were collected and subsequently the oil-soaked batting could be weighed to determine the mass of unburned oil.

Soot samples were collected on quartz fiber filters for thermal-optical analysis of elemental versus organic carbon content. Standard precautions were taken to avoid sample contamination before sample collection by heating the filter for several hours at 700 °C and also heating the aluminum foil used to line the sample containers to 500 °C for several hours. The second collection system was used for the quartz filters, but the PUF samples were not collected. Because the thermal-optical technique requires much less soot per filter, smoke sampling flow rates averaged 2 L/min.

##### 4.2.3.2 PAH and Carbon Quantification

The PAH analyses of 23 Teflon filters and 12 PUF samples were performed by the Environment Canada (EC) at Ottawa and the Chemical Science and Technology Laboratory at NIST. The analyses at NIST involved gas chromatography (GC) with flame-ionization detection, while EC used GC with mass spectrometric detection (GC-MS) for quantifying the individual PAHs. In addition, PAH analyses were performed on five crude oil samples and seven burn residue samples.

The Teflon filters and PUF samples analyzed at NIST were spiked with appropriate amounts of an internal standard containing phenanthrene-d_10_ and 1-n-butylpyrene and then Soxhlet extracted with dichloromethane (DCM) for 13 h to 18 h. A response/recovery solution containing known amounts of 17 PAHs was spiked and processed in the same manner as the samples. The details of the sample handling including the concentrating of the sample, the extraction cartridges, and chromatographic columns are described in a separate publication [23].

The crude oil samples and residues that were analyzed at NIST were prepared as described previously by Kline et al. [40]. Briefly, the samples were weighed into 50 mL Erlenmeyer flasks, spiked with the same internal standard solution described above, diluted with 2 mL of a 5 % DCM in pentane, and manually agitated for 1 min. A PAH fraction for the crude oil and residue samples as well as a response/recovery solution were collected in the same way as for the filter and PUF extracts. Silica cartridges were not used in preparing the crude oil and residue samples for the liquid chromatography (LC) fractionation procedure.

Gas chromatography with flame-ionization detection was used at NIST to measure PAHs in the LC fractions of the filter, PUF, and crude samples. Again, the details of operating parameters for the GC-FID are given in a separate publication [23]. The Teflon filter samples were run in duplicate, while the PUF, crude oil, and residue samples were analyzed once. The two pieces of foam making up sample PUF-12 (from the phase II experiment) were extracted separately to determine whether or not any of the PAHs were collected on the downstream portion of PUF. The presence of PAHs on both segments would suggest breakthrough of these species on the PUF.

The samples analyzed at EC were spiked with a mixture of deuterated PAHs and extracted in DCM in the dark for approximately 14 h. For the oil sample, 0.75 g was dissolved in 3.75 mL of cyclohexane and an aliquot equivalent to 0.08 g of oil was used for cleanup. The raw extract was fractionated on a silica column. The benzene fraction, containing the PAHs, was concentrated to 0.5 mL and analyzed by GC-MS using a five-step selected ion monitoring (SIM) program and the same type of GC column as that used in the NIST study. A mixture of PAH standards was processed at the same time to assess PAH recovery and response. While extensive preseparation was necessary to isolate the PAH-rich fraction when gas chromatography with flame-ionization detection was used at NIST, mass spectrometric detection used at EC required a minimal amount of preparation.

Although many PAHs were identified, PAHs were only one component of the so-called organic carbon fraction. The total organic carbon fraction was determined by a contract laboratory using thermo-optical analysis for the organic/elemental carbon [41] of particulate collected on quartz fiber filters.

##### 4.2.3.3 PAH Results

In general, the Teflon filter samples collected at ambient temperature had significantly higher concentrations of all the PAHs determined; while the samples collected at the higher temperature were biased toward the higher molecular weight compounds because the majority of the three- and four-ringed compounds were present in the vapor phase and were not retained on the filters. Concentrations of 18 PAHs were determined (Table 3) in the four filter extracts from high-temperature samples and four filter extracts from low-temperature samples during the phase I experiment. In the phase I study the particulate samples collected at high temperature were sequential, and the samples collected during the latter part of the burn, such as H2 #2, analyzed at NIST, and H4 #2, analyzed at EC, appear to be enriched in the larger, lower vapor pressure PAHs.

Since the high-temperature samples were collected over a short portion of the burn, the high-temperature results of the two laboratories should not be directly compared. However, for the cold samples, the smoke was collected during the entire burn so that the results are comparable and the agreement between the two laboratories is typically 10 % to 20 % for the individual PAHs for samples C5 (NIST) and C7 (EC).

**Fig. 23:**
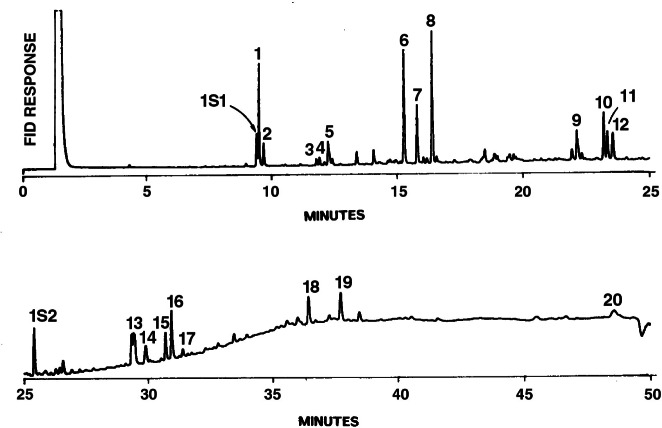
Gas chromatogram on Teflon filter sample T-12. Peak identifications: (ISI) phenanthrene-d_10_; (1) phenathrene; (2) anthracene; (3) 2-methylphenathrene; (4) 3-methylphenanthrene; (5) 1-methylphenathrene; (6) fluoranthrene; (7) acephenathrylene; (8) pyrene; (9) benzo[*ghi*]fluoranthene; (10) cyclopenta[*cd*]pyrene; (11) benz[*a*]anthracene; (12) chrysene and triphenylene; (IS2) 1-*n*-butylpyrene; (13) benzo[*b*, *j*, and *k*]fluoranthenes; (14) benzo[*a*]fluoranthene; (15) benzo[*e*]pyrene; (16) benzo[*a*]pyrene; (17) perylene; (18) indeno[1,2,3-*cd*]pyrene; (19) benzo[*ghi*]perylene; (20) coronene.

A gas chromatogram of a typical crude oil combustion sample (T-12) is shown in Fig. 23. The gas chromatogram of a spiked field blank shows little response except for one contaminant, which did not affect the PAH analysis. These crude oil combustion samples displayed the entire distribution of PAHs typical for soot samples, from the three-ring phenanthrene and anthracene to the seven-ringed coronene.

Analyses of PAHs in the two crude oil and three residue samples from the phase II experiments (Table 4) indicate their PAH concentrations were comparable. Over 90 % of the PAH content in these samples consists of three-membered-ring PAHs with high concentrations of phenanthrene, methylphenanthrenes, and dimethylphenanthrenes. The concentrations of four- and five-ring PAHs were less than the three-membered-ring compounds by a factor of 10 or more. The soot samples were noticeably depleted in the alkylphenanthrenes compared with the crude oil and residue samples, suggesting that the soot samples do not contain large amounts of unburned crude oil (< 10 %).

The organic carbon fraction of the smoke was in the range of 14 % to 21 % without any significant trend with oil layer thickness. The remaining 79 % to 86 % was classified as elemental carbon. These results are to be compared with organic carbon fractions of 10 % or less obtained from the collection of smoke in phase I from the steady-burning phase. The higher values of organic carbon observed in phase II may be the result of fuel droplets being emitted from the surface and collected during the rapid-boiling phase.

##### 4.2.3.4 PAH Discussion

In general the Teflon filter samples collected at the lower temperature had significantly higher concentrations of all the PAHs determined. They would be expected to better represent samples collected downwind of an ambient air crude oil burn, given the cooling that the smoke experiences upon dilution. Particulate- and vapor-phase sample analysis demonstrated that a majority of the three-ring PAHs were collected in the vapor phase. There was an indication that desorption of three-ring PAHs from the particulate was taking place.

The burning of crude oil produced less total PAHs (sum of the amounts in the residue and in the filter and PUF samples) than were in the original crude oil (Table 5). While about 90 % of the PAHs for the oil and residue had three rings, the PAH content of the combustion emissions (particulate and vapor) was distributed equally among three-ring and larger compounds (Table 4). The concentrations of PAHs with five or more rings, which includes benzo[a]pyrene, were 10 to 20 times greater in the smoke than in the original fuel. The adverse affects of generating higher levels of carcinogenic PAHs such as benzo[a]pyrene must be weighed against the benefits of in situ burning: the reduction of local damage by consuming much of the spill, the reduction in the amount. of PAHs in the water, and the dispersion of the smoke and PAHs over a much larger area.

To provide a perspective on the PAH emission from the burning of crude oil, it is useful to compare the present results with the PAH emissions from wood-burning stoves. There have been a large number of studies on PAH emissions from stoves [42 43 44 45 46 47] and the results vary from a low of 5 µg to 10 µg of PAHs/g of wood consumed to a high of 200 µg to 400 µg of PAHs/g of wood consumed. The higher values are correlated with decreased air supply and increased fuel loading. Assuming a value of 50 µg of PAHs/g of wood consumed, which is about 8 times less than for crude oil, a nominal burning rate of 4 kg/h, and a 12 h burn time, one estimates a total PAH emission for one stove of 2.4 g/day. This would correspond to the burning of 7 L of crude oil, assuming an emission of 400 µg of PAHs/g of crude oil (Table 4). Scaling up this model, one finds that the PAHs emitted from burning a 260 000 L crude oil spill would equal the amount produced by 50 000 wood-burning stoves. There is a large uncertainty in the number of stoves in this comparison (or number of liters in the first comparison), first because of the wide range of burning conditions for the stoves including stove design, type of fuel, fuel loading, and amount of air, and second because the PAH emission from the crude oil may be affected by the wind, sea conditions, and size of the fire. It should be noted that the actual human exposure from either burning event would be heavily dependent on the meteorological conditions (e.g., wind direction and speed, thickness of the inversion layer, etc.) during the episodes. Of course, one must have a quantitative smoke-dispersion / deposition model for assessing the downwind impact of the smoke.

## 5. Smoke Plume Trajectory Computations

### 5.1 Smoke Plume Dispersion by Atmospheric Winds

Buoyant windblown plumes have been studied since the early 1960s. A summary of the early work together with a useful bibliography is given by Turner [48]. For summaries of more recent work see Turner [49] and Wilson [50], as well as actual “User’s Guides” for some of the more popular models [51, 52, 53, 54]. Virtually all the models described in these works are integral models, where the profiles of physical quantities in cross-sectional planes perpendicular to the wind direction are assumed, together with simple laws relating entrainment into the plume to macroscopic features used to describe its evolution. A great many of the models in use for air quality assessment simply use Gaussian profiles of pollutant density. However, the plume structures actually observed are often too complex to be described in terms of a few simple parameters. This is especially true of plumes lofting over complex terrain.

Most of the assumptions required by integral models can be removed by taking advantage of the enormous advances in computational fluid dynamics that have occurred since most of these models were developed. Of course, large scale computations of atmospheric phenomena can still overwhelm even the fastest computing platforms, but by applying some reasonable approximations to the equations governing the fluid flow, it is possible to reduce the size of the computations to fit onto a reasonably priced personal computer. One particularly useful approximation for the windblown plume problem is to assume that the component of the fluid velocity in the direction of the ambient wind is literally the wind speed. The neglect of streamwise perturbations to the ambient wind is an old idea in aerodynamics, where it has been used to study aircraft wake dynamics since the 1930s [55]. Once this approximation is made, the plume (or wake) can be studied as a two-dimensional, time-dependent entity. The large scale structure of the plume can then be determined in detail at moderate computational cost.

This approach was first used to study the settling of a smoke plume in an unstratified atmosphere in a NIST funded collaboration with Massachusetts Institute of Technology (MIT) researchers, see Ghoniem et al. [56]. This study was performed using Lagrangian vortex dynamics techniques. The main emphasis was on the mixing process as it affected the plume structure.

#### 5.1.1 ALOFT Model

The ALOFT (A Large Outdoor Fire plume Trajectory) model developed at NIST takes an approach similar to that of Ghoniem et al [56], but it uses finite-difference methods to determine the large scale mixing, combined with a Lagrangian description of the transport of the smoke and other pollutants. The effect of sub-grid scale velocity fluctuations on the dispersion of the smoke is accounted for explicitly, and the ambient temperature profile is subject only to the constraint that it is stable over the altitudes occupied by the plume.

The ALOFT model consists of the conservation equations of mass, momentum and energy that describe the steady-state convective transport of heated gases introduced into the atmosphere by a steadily burning fire. The fire itself is not modeled; the smoke plume is the main interest. The fire is represented as a source of heat and smoke, but not necessarily as a point source. Only the overall fuel consumption and heat release rates per unit burn area, plus the fuel-specific emission factors for the combustion products of interest, need be specified. This information is all derived from experimental measurements. The local meteorological conditions that must be provided are the wind speed, the magnitude of the fluctuation of the wind from the prevailing direction, and the temperature stratification of the atmosphere. Because the model is based on the fundamental conservation equations and does not rely on empirical correlations to describe the plume rise and dispersion, additional physical phenomena can be included in the model if necessary.

The development of the ALOFT model began in the early 1990s. The intent of the effort is to solve a simplified form of the equations of motion that govern the introduction of smoke and hot gases from a large fire into the atmosphere. It is assumed that the smoke plume is blown by a non-zero wind over relatively flat terrain (i.e., the sea surface or a flat coastal area). This version of the model is now referred to as ALOFT-FT (Flat Terrain) [57,58]. The flat terrain assumption is crucial, for it leads to the assumption that the windward component of the flow of smoke and hot gases from the fire *is* the prevailing wind, and the numerical problem is reduced to solving for the fire-induced components of velocity and temperature in a plane perpendicular to the prevailing wind. From a computational point of view, this simplifies the problem tremendously and allows for well-resolved computations of the plume dynamics as it rises and levels off in the atmosphere. High resolution in this case refers to the fact that motion on length scales of 5 m to 10 m is captured directly.

The ALOFT model differs from most of the atmospheric dispersion models in use today because it is a *deterministic* rather than an *empirical* model. The approach is to solve the equations governing the flow rather than to rely on empirical formulae that approximate the extent of the dispersion. Empirical models typically assume the pollutant is Gaussian-distributed in the plane perpendicular to the direction of the prevailing wind. The parameters defining the distribution are estimated from experiments. However, Gaussian models are inappropriate for two reasons: (1) the characteristics of the “source” are different from the smokestacks that are usually assumed by such models, and (2) the size of the source is well beyond those considered in industrial applications and thus outside of the experimental parameter range used to calibrate the models.

The rise of a smoke plume from a large fire is governed by the complicated mixing of the hot combustion products with the surrounding air, a process known as entrainment. The extent to which the hot gases are cooled and diluted by the entrained air determines how high the plume will rise. The fires considered in this study generate hundreds of megawatts of energy, and the smoke plumes can rise a few hundred meters to a few kilometers into the atmosphere, depending on the temperature stratification. Often, conventional dispersion models characterize the source in terms of an exit velocity and temperature. Even if a characteristic velocity and temperature of the hot gases near the fire could be ascertained, there is no way to accurately determine the ultimate height to which the plume will rise unless a calculation is performed that explicitly accounts for the mixing processes. This is especially true of atmospheres exhibiting non-linear temperature profiles and features such as temperature inversions.

The ALOFT model exploits the tremendous power of modern computers to solve a simplified version of the Navier-Stokes equations that govern the convective mixing processes. These equations express the conservation laws of mass, momentum and energy of the hot combustion gases as they mix with the atmosphere. Because of the fundamental nature of the governing equations, far fewer empirical parameters need be input by the user. Ultimately, this simplification will prove to be the most beneficial improvement offered by this direct approach. As the problems of atmospheric dispersion become increasingly complicated by the addition of more physical phenomena, the number of empirical model input parameters will increase tremendously, but the amount of available field data will remain limited due to the difficulty of conducting such experiments. Indeed, this is why numerical models were developed in the first place. With a lack of good data to calibrate empirical models, especially in cases involving complex terrain, the direct approach of solving the fundamental equations of motion has become more attractive.

### 5.2 The Smoke Plume

The plume is described in terms of steady-state convective transport by a uniform ambient wind of heated gases and particulate matter introduced into a stably stratified atmosphere by a continuously burning fire [57,58]. Since the firebed itself is not the object under study, only the overall heat release rate and the fraction of the fuel converted to particulate matter need be specified. The simulation begins several fire diameters downwind of the fire, where the plume is characterized by relatively small temperature perturbations and minimal radiation effects. In this region the plume gases ascend to an altitude of neutral buoyancy, and then gradually disperse. The trajectory of the plume is governed by the ambient wind, the atmospheric stratification and the buoyancy induced convection. It is assumed that the ambient temperature profile as a function of height is available. The model has been extended to allow for multiple interacting plumes [59] and the presence of a wind shear [60]. However, only the basic form of the model will be discussed in detail here.

Assuming that the perturbations to the background temperature *T*_0_(*z*) and pressure *P*_0_(*z*) are small beyond a few diameters downwind of the firebed, the expansion component of the velocity field can be ignored and the equations describing the steady-state plume reduce to the Boussinesq approximation. The uniform ambient wind speed *U* is taken to be constant. For mathematical consistency, *U* is much larger than the buoyancy induced crosswind velocity components, and the rates of change of physical quantities in the windward direction are much slower than those in the crosswind plane. These assumptions are quite realistic several flame lengths downwind of the firebed. Since *U* does not change, there is no need for a windward component of the momentum equations. The details of the firebed are not being simulated, so the only information about the fire required is the overall convective heat release rate *Q*_0_ and the particulate mass flux. The initial temperature distribution in the plume cross section is assumed to be Gaussian and satisfy the following integral

$$\int_{-\infty }^{\infty }\int_{0}^{\infty }\rho_{0}c_{p}U\tilde{T}dzdy=Q_{0}$$

where the quantity $\tilde{T}$ is the fire induced temperature perturbation. The particulate matter (or any non-reacting combustion product) is tracked through the use of Lagrangian particles which are advected with the overall flow. The initial particulate distribution mimics the initial temperature distribution. If either more detailed experimental data or the results of a local simulation of the firebed dynamics is available, then these could be used in lieu of the Gaussian profile.

The equations of motion are made non-dimensional so as to maximize the amount of information which can be extracted from each run. First, the windward spatial coordinate is replaced by a temporal coordinate

$$t*=\frac{V}{UL}x$$

where the plume height *L* is given in terms of the potential temperature of the undisturbed atmosphere *θ* (*z*)

$$L={\left(\frac{Q_{0}}{C_{p}T_{0}\rho_{0}U\theta^{\prime }}\right)}_{z=0}^{1/3};\theta^{\prime }\left(z\right)=\frac{1}{\theta }\frac{d\theta }{dz}.$$

The potential temperature is related to the actual temperature through the relation

$$P_{0}^{-\kappa }\left(z\right)T_{0}\left(z\right)=P_{0}{\left(0\right)}^{-\kappa }\theta \left(z\right)$$

where *κ* = *R*/*C*_p_ and *R* is the gas constant for dry air. The characteristic velocity of the fluid is given by

$$V={\left(\frac{Q_{0}g}{C_{p}T_{0}\rho_{0}UL}\right)}_{z=0}^{1/2}.$$

The characteristic length *L* and velocity *V* scale the crosswind spatial coordinates (*y*, *z*) = *L*(y*, z*) and velocities (*v*, *w*) = *V*(v*, w*). The quantity *θ′* (*z*) is scaled by its value at the ground. The temperature perturbation $\tilde{T}$ is made nondimensional by the expression

$$\tilde{T}=\left(\frac{Q_{0}}{C_{p}\rho_{0}UL^{2}}\right)T*.$$

Finally, the turbulent Reynolds and Prandtl numbers are defined

$$Re=\frac{\rho_{0}VL}{\mu };Pr=\frac{\mu C_{p}}{k}.$$

The viscosity and thermal conductivity are to be regarded as “eddy” coefficients whose primary role is to provide sinks of kinetic and thermal energy that are actually the result of sub-grid scale dissipative processes. In practice, they are used to set the dynamic range of length scales employed in the simulation, which is typically 5 to 15 meters. This range is needed to capture the large-scale fire induced eddy motions. This requirement, together with the knowledge that the dissipative effects operate at a scale *Re*^−1/2^ times smaller than the overall geometric scale (the stablization height of the plume for this problem), translates into Reynolds number of the order 10^4^. Thermal conductivity is treated in a similar manner to viscosity; thus the Prandtl number remains or order unity.

The dimensionless form of the model equations is remarkably simple

$$\begin{array}{l} \frac{\partial v*}{\partial y*}+\frac{\partial w*}{\partial z*}=0\\ \frac{\partial v*}{\partial t*}+v*\frac{\partial v*}{\partial y*}+w*\frac{\partial v*}{\partial z*}+\frac{\partial p*}{\partial y*}=\frac{1}{Re}\left(\frac{\partial^{2}v*}{\partial y{*}^{2}}+\frac{\partial^{2}v*}{\partial z{*}^{2}}\right)\\ \frac{\partial w*}{\partial t*}+v*\frac{\partial w*}{\partial y*}+w*\frac{\partial w*}{\partial z*}+\frac{\partial p*}{\partial z*}-T*\\ \;=\frac{1}{Re}\left(\frac{\partial^{2}w*}{\partial y{*}^{2}}+\frac{\partial^{2}w*}{\partial z{*}^{2}}\right)\\ \frac{\partial T*}{\partial t*}+v*\frac{\partial T*}{\partial y*}+w*\frac{\partial T*}{\partial z*}+\theta *{\left(z\right)}^{\prime }w*\\ \;=\frac{1}{Re\;Pr}\left(\frac{\partial^{2}T*}{\partial y{*}^{2}}+\frac{\partial^{2}T*}{\partial z{*}^{2}}\right). \end{array}$$

subject to the initial condition

∬T*(y*,z*)dy*dz*=1.

The crosswind velocity components v* and w* are assumed to be zero initially, although this assumption can be relaxed if more detailed information is available. No-flux, free-slip boundary conditions are prescribed at the ground, consistent with the assumed uniformity of the prevailing wind and the resolution limits of the calculation. At the outer and upper edges of the computational domain, the perturbation temperature, perturbation pressure, and windward component of vorticity are set to zero.

**Fig. 24:**
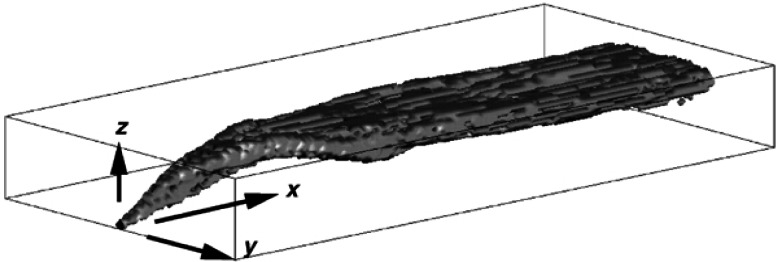
Three dimensional view of a computed smoke plume in the first few kilometers of its development. The height of the viewbox is 1 km, the length 8 km, and the crosswind length 4 km. The wind speed is 6 m/s. The computation is initialized by prescribing the temperature and particulate distribution in the plane spanned by the *y* and *z* coordinates. Then the plume is constructed as the initial plane is swept downwind.

**Fig. 25:**
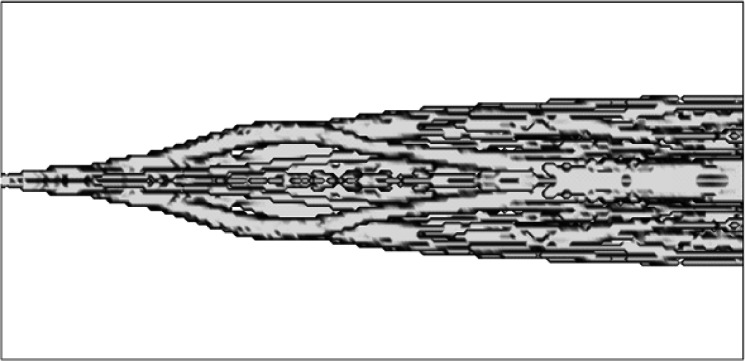
A view of the plume from below. Note the separation and reconnection of the two large counter-rotating vortices.

**Fig. 26:**
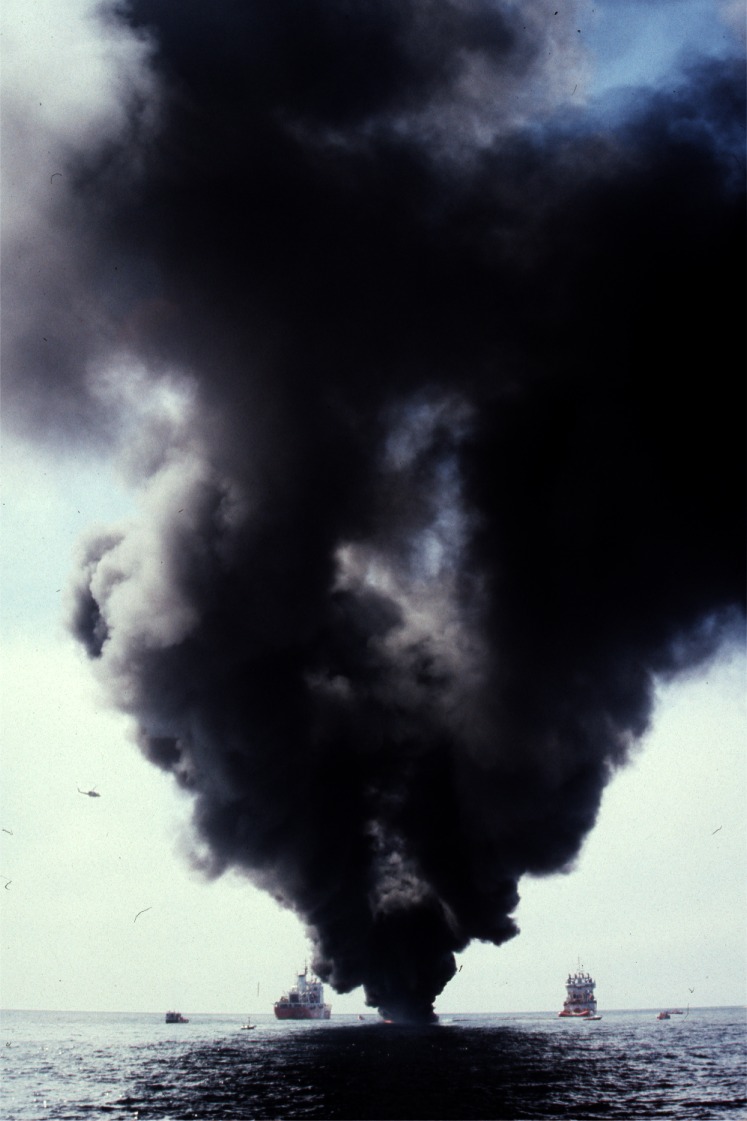
Photograph taken from about 200 m downwind of the Newfoundland Offshore Burn Experiment (NOBE) showing the two large counter-rotating vortices which characterize the structure of the rising smoke plume.

Figures 24 and 25 show the results of a sample computation, illustrating the position of the initial slice and the extent of the computational domain. The plume is visualized by interpolating the particle locations onto the computational grid, and then plotting the isosurface on which the particulate density is zero. Figure 25 shows the plume from underneath, illustrating the structure of the large, counter-rotating vortices that are generated by the rising plume. This vortex structure is a dominant feature of the rising plume, and governs the rate at which fresh air is mixed in with the hot combustion products. Figure 26 is a photograph taken about 100 m downwind of the Newfoundland Offshore Burn Experiment, and it shows clearly the development of the two vortices. An excellent discussion of these structures is given in Ref. [61].

The solutions to the Boussinesq equations described above may be regarded as “time-averaged”. The trajectories of the Lagrangian particles used to represent the smoke particulate are randomly perturbed from their mean paths in order to mimic the spatial and temporal fluctuations of the wind and the underlying turbulence. Specifically, the motion of each particle is governed by the mean wind field (*u*,*v*,*w*) plus a perturbation velocity field (*u′*,*v′*,*w′*) that represents the random temporal and spatial variations of the ambient wind. For simplicity, it will be assumed that the wind direction is aligned with the velocity component *u*, even though the numerical algorithm does not require the wind to be aligned with the *x* coordinate. Most meteorological texts adhere to the convention that *v* and *w* are perpendicular to the direction of the prevailing wind. Indeed, this is the case for the two-dimensional form of the equations. The perturbation velocity components are derived from the recursive relations

$$\begin{array}{l} u^{\prime }\left(t+\delta t\right)=R_{u}\left(\delta t\right)u^{\prime }\left(t\right)+u^{\prime \prime };\;R_{u}\left(\delta t\right)=e^{-\delta t/\tau_{u}}\\ v^{\prime }\left(t+\delta t\right)=R_{v}\left(\delta t\right)v^{\prime }\left(t\right)+v^{\prime \prime };\;R_{v}\left(\delta t\right)=e^{-\delta t/\tau_{v}}\\ w^{\prime }\left(t+\delta t\right)=R_{w}\left(\delta t\right)w^{\prime }\left(t\right)+w^{\prime \prime };\;R_{w}\left(\delta t\right)=e^{-\delta t/\tau_{w}}. \end{array}$$

The double-primed terms are random variables with Gaussian distributions whose variances are that of the perturbation velocities multiplied by (1−*R_u_*^2^), (1−*R_v_*^2^) and (1−*R_w_*^2^), respectively, ensuring that the variance of the each velocity component will not change from one time step to another. The variance of *v′* and *w′* are denoted in the literature as *σ_v_*^2^ and *σ_w_*^2^, respectively. The fluctuation of the windward velocity component *u* can be associated with wind gusts. The functions *R_u_*, *R_v_*, and *R_w_* are Lagrangian correlation coefficients, taken as exponentials. The parameter *τ* is indicative of the period of atmospheric fluctuations. Appropriate values for various meteorological conditions are given by Draxler [62]. Generally speaking, *τ* is on the order of several minutes.

A popular classification scheme for defining the turbulence of the atmosphere is given by Pasquill [63]. Corresponding to each stability category are values for the standard deviation of the prevailing wind direction in the horizontal and vertical directions. Reference [64] contains a discussion of these parameters and methods of evaluating them. Usually, the smoke plume resides mainly in what is referred to as the planetary boundary layer (PBL). Sometimes this region is also called the mixing layer, although the precise definitions of these terms varies depending on the specific application. For the discussion to follow, the boundary or mixing layer is that part of the troposphere that is directly influenced by the presence of the earth’s surface. The depth of this layer can vary from roughly 50 meters to several thousand meters. Within it, the interaction of the complex terrain, solar heating and surface friction creates a turbulent wind field, to which the solution of the above equations may be considered a time-average. The values of the wind fluctuation parameters given above are appropriate within this mixing layer. However, it often happens that the smoke plume, due to the tremendous thermal buoyancy, will penetrate the top of the mixing layer. When this happens, the plume is subject to far less turbulent motion because the air currents are more representative of the free atmosphere. As a result, the magnitude of the wind fluctuations used in the model are reduced for those particles that penetrate the top of the mixing layer.

Finally, it should be noted that the model of atmospheric turbulence discussed in this section is relatively simple. There exist in the literature more elaborate models, and the user is directed to any number of references that provide correlations based on various other observed conditions [52, 54, 63, 64]. The best source of wind fluctuation parameters is an anemometer, but this type of data is often hard to come by for a given region and a given set of atmospheric conditions.

### 5.3 Validation Experiments for ALOFT-FT

The model predictions were compared with measurements taken at three field experiments. The following sections document the comparisons. It should be pointed out that the experimental data was used to assess the accuracy of the model predictions. The data was *not* used to calibrate the model. This is an important distinction, and it points out the difference between a deterministic and an empirical model.

#### 5.3.1 The Newfoundland Offshore Burn Experiment (NOBE)

The Newfoundland Offshore Burn Experiment (NOBE) provided an enormous amount of data regarding in situ burning of oil at sea. The experiment consisted of two burns of crude oil conducted off the coast of St. John’s, Newfoundland on August 12, 1993. Most of the sampling of the chemical species produced by the burning was done relatively close to the fire. However, the University of Washington’s Cloud and Aerosol Research Group performed airborne measurements of the smoke plume from the two burns at distances up to 20 km downwind of the fire. Of particular importance to the present study are the lidar (Light Detection and Ranging) measurements of the plume cross section, and the real-time monitoring of the CO_2_ level in the plume.

**Fig. 27:**
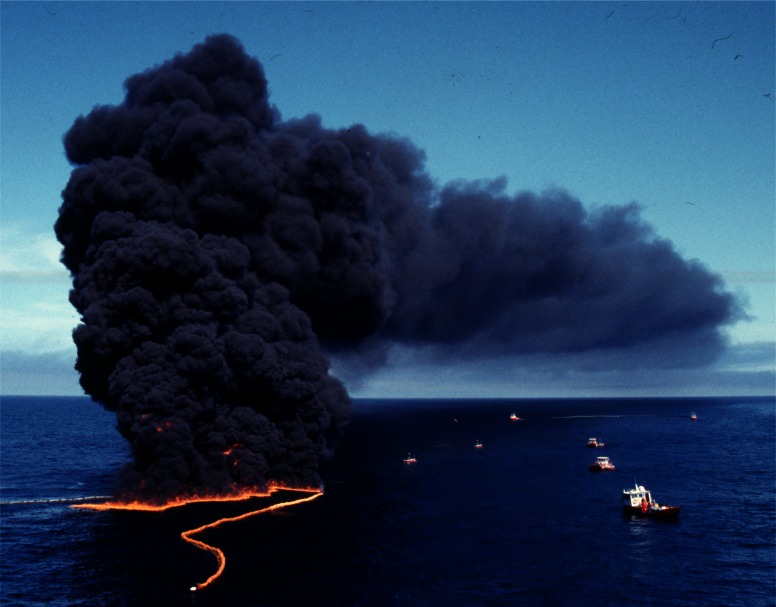
Photograph of the Newfoundland Offshore Burn Experiment showing the shift of the wind at about 120 m off the surface.

Lidar measurements were performed during the second burn. For this burn, it was reported that 28.9 m^3^ of Alberta Sweet Mixed Blend crude oil of density 843.7 kg/m^3^ was burned in 1.3 h [65]. Even though substantial fluctuations in burning rate were observed, for the purposes of modeling the plume it was assumed that the burning rate was constant at 5.2 kg/s. Based on previous work with Louisiana crude [11], the effective heat of combustion of the oil was assumed to be 42 000 kJ/kg, even though a different oil was used for the experiment. The smoke yield for the burn was measured by the team from NIST to be approximately 15 % [30], and the fraction of the total heat release lost from the flame as radiation through the dense smoke plume was assumed to be 10 % [66]. Thus, the *convective* heat release rate for the model run was about 200 MW and the particulate production rate was 0.78 kg/s. Atmospheric temperature soundings taken from the University of Washington airplane [67] and from the NIST tethered blimp [66] show a temperature inversion from about 100 m to 175 m in altitude, accompanied by a shift of roughly 30° to 40° in the direction of the wind. This shift in the wind can be seen in the photograph presented in Fig. 27. The wind speed at the ground was about 5 m/s to 6 m/s, increasing to about 8 m/s a few hundred meters up.

**Fig. 28:**
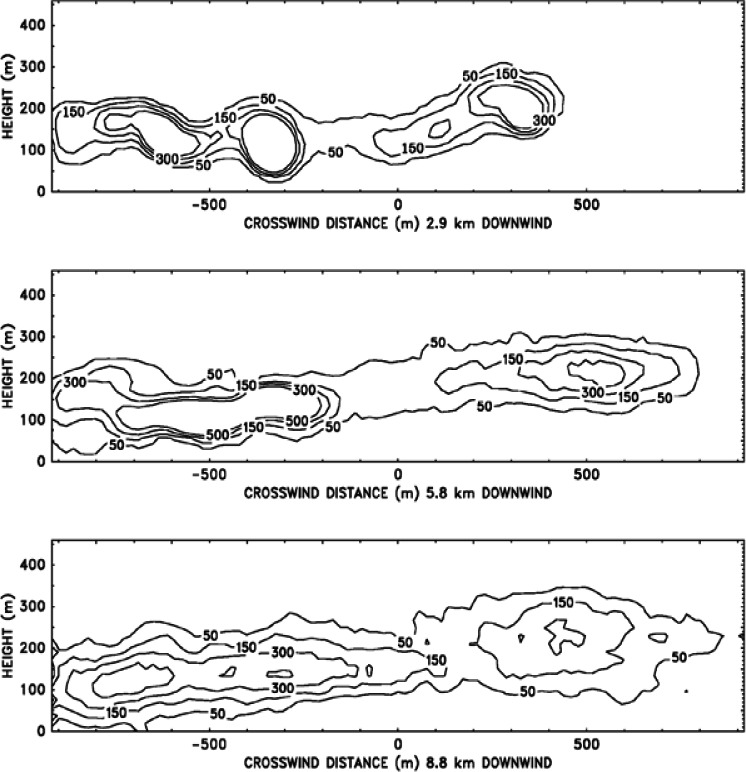
Time-averaged cross sectional slices of the simulated smoke plume from the second NOBE burn. Shown are particulate concentration contours of (50, 150, 300, and 500) µg/m^3^ at three locations downwind corresponding to where lidar measurements were taken. The vertical length scale indicates height above sea level, while the horizontal scale indicates the distance from the assumed plume centerline.

**Fig. 29:**
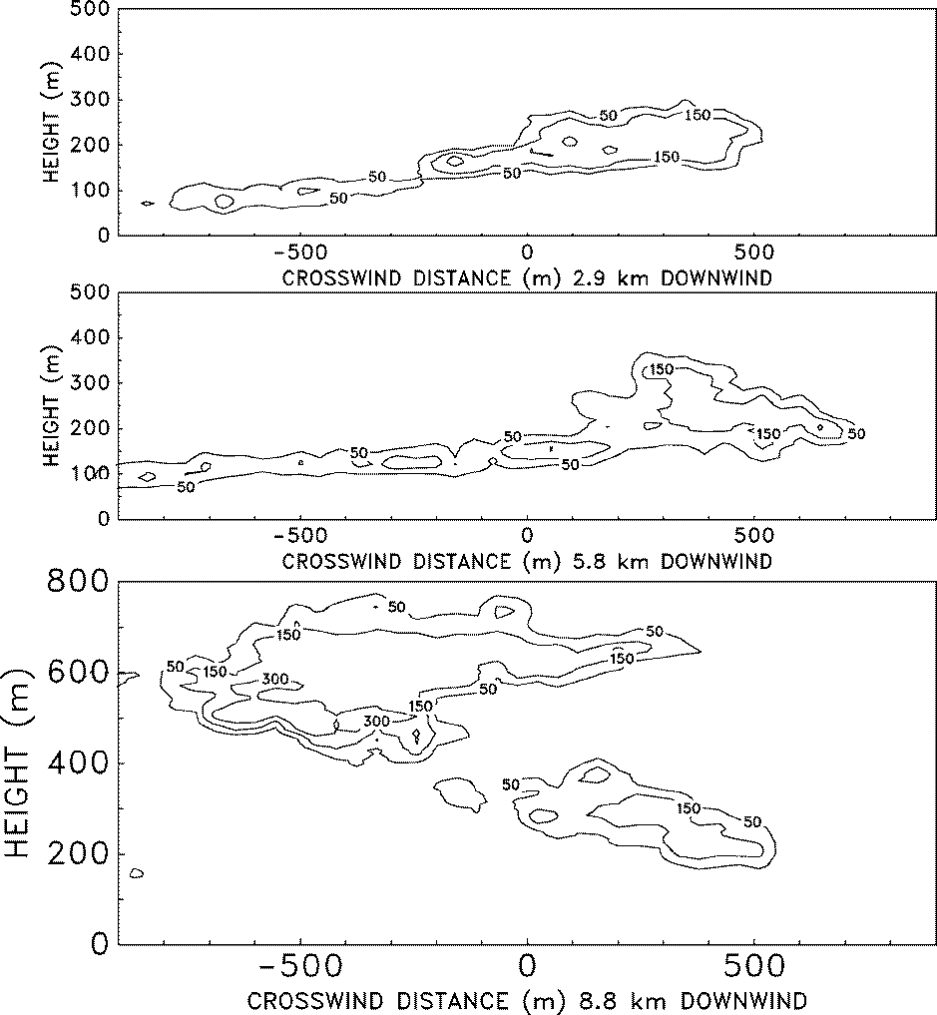
Instantaneous cross sectional slices of the actual smoke plume from the second NOBE burn, courtesy of the University of Washington Cloud and Aerosol Research Group. Shown are contours of particulate concentration at (50, 150 and 300) µg/m^3^. The crosswind scale indicates relative distances, and the origin was chosen to compare with the simulation.

Figure 28 displays time-averaged cross sections of the simulated plume at downwind locations comparable to those at which lidar measurements were made from the University of Washington aircraft (see Fig. 29). The shift in the wind direction at about 120 m in altitude dramatically increases the lateral width of the plume, spreading the smoke over a 2 km wide path. This spreading is seen in both the simulated and the actual plume cross sections. There is qualitative and quantitative [58] agreement between the two for a distance of about 6 km from the fire. This assessment is based mainly on the height and lateral extent of the simulated plume in comparison to the lidar images. It should be emphasized that the lidar images reflect the instantaneous plume cross section, whereas the simulated cross sections represent a time-averaged picture.

**Fig. 30:**
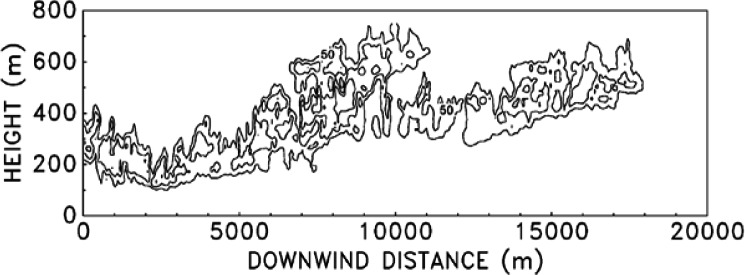
Lidar measurement of plume centerline of the second burn taken from University of Washington airplane. Note that the horizontal and vertical length scales are very different. In actuality, the plume shown is a long, slender object. Also, the origin of the plot is about 0.9 km from the actual fire.

Beyond 6 km from the fire the numerical model does not predict the additional lofting of the plume shown by a lidar trace along the approximate plume centerline (Fig. 30). The model correctly predicts the initial rise height of 200 m, but after about 6 km, the plume gradually rises to a height of about 600 m. The centerline of the simulated plume reaches a height of about 250 m, but does not exhibit this gradual rise. It is unclear exactly why it occurs. It has been speculated that this lofting might be due to the heat generated by the absorption of sunlight by the smoke particulate. Another explanation is the possible presence of local convective cells in the path of the plume. These updrafts occur over small areas and cannot be predicted from the meteorology of the entire region. In any case, this example points out the limitation of any predictive dispersion or meteorological model. Large scale patterns and trends can be predicted, but small-scale details cannot.

In addition to lidar measurements, the University of Washington airplane made a number of other measurements. Of interest to this study are measurements of CO_2_. Plume particulate concentrations may be derived either by quantifying lidar cross section data as shown above, or by measuring the excess CO_2_ and backing out the particulate concentration based on the smoke yield and the elemental carbon mass fraction of the fuel. Direct measurements of excess CO_2_ made while flying the airplane along the centerline of the plume have been used to estimate the concentration of particulate matter. Taking the smoke yield to be 15 % (from the NIST tethered blimp) and the elemental carbon mass fraction of the fuel to be 0.8664; it is estimated that a volume fraction of 1 × 10^−6^ of CO_2_ in excess of the ambient air value corresponds to a particulate concentration of 103 µg/m^3^. Direct measurements of excess CO_2_ from the airplane show volume fractions decreasing to about 1.5 × 10^−6^ (the equivalent of 150 µg/m^3^ particulate) by about 16 km downwind of the burn. The quantified lidar images are consistent with this finding. The model calculation predicts that concentrations in excess of 150 µg/m^3^ extend slightly farther than 20 km downwind. The discrepancy in the two estimates is not surprising, given the enhanced plume dispersion of the experiment due to the unexpected lofting. Also, the comparison is being made based on only one pass of the airplane along the plume centerline, which may not account for the maximum concentration. Indeed, the model predicts, and visual sightings confirm, the existence of counter-rotating vortices which are generated by the fire and which entrain a substantial fraction of the particulate. Thus, it is not necessarily true that the maximum concentration of particulate would be found along the centerline of the plume. In situ measurements of the plume cannot account for its complex structure, and thus a better means of measuring particulate concentration would be through the use of integrated techniques, such as the lidar measurements discussed above.

#### 5.3.2 Alaska Clean Seas Burning of Emulsions Experiment

**Fig. 31:**
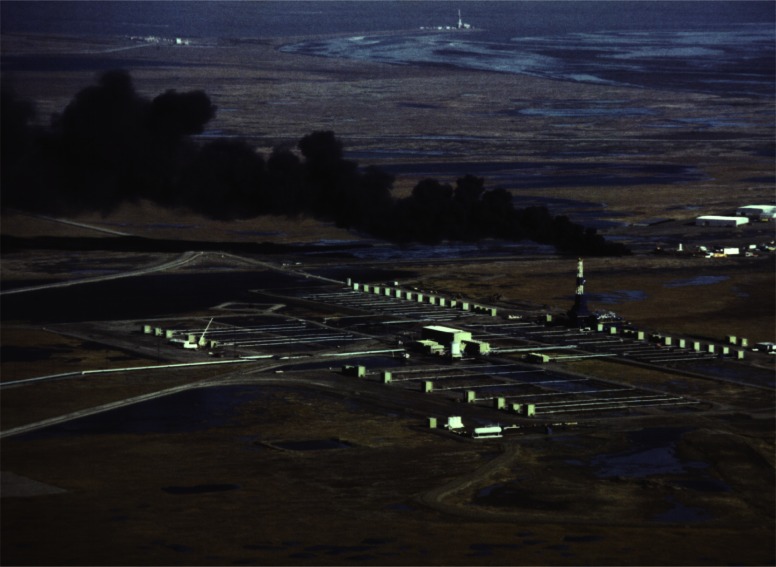
Aerial photograph taken of the second ACS burn, Prudhoe Bay, September 1994.

In early September 1994, Alaska Clean Seas conducted at its Fire Training Ground in Prudhoe Bay, Alaska, three mesoscale burns to determine the feasibility of burning emulsified oil [68]. Fig. 31 shows an aerial view of the second burn. At the request of the Alaska office of the US Environmental Protection Agency, the EPA’s Emergency Response Team (EPA/ERT) came to Prudhoe Bay with 12 real-time aerosol monitors (RAMs). These instruments use a light scattering technique to measure particulate concentrations.

The twelve instruments were set out on meter high tripods, spread out in rows of three or four, at distances ranging from 1 km to 5 km from the burn site. The deployment strategy varied from burn to burn, depending on the weather conditions and the terrain over which the plume was expected to loft. The instruments were set to sample every second, and then log the 5 s average. Global positioning instruments recorded the locations of the individual devices.

Table 6 summarizes the three mesoscale emulsion burns. Each burn consisted of burning an oil mixture within the confines of a fire-resistant circular boom which floated in a pit filled with water. The boom diameter was roughly 9 m, and the rectangular pit was roughly 20 m by 30 m. The first and third burns consumed emulsions of salt water and 17.4 % evaporated Alaska North Slope crude. Emulsion breakers were applied to these mixtures. The second burn consumed fresh ANS crude. To compute the average heat release rate for the burns, the mass of oil consumed (Oil Mass × Removal Efficiency) was multiplied by a total heat of combustion of 42 000 kJ/kg, and then divided by the number of seconds needed to consume the oil. As an input to the ALOFT model, an estimate is made that 90 % of the total heat release rate may be considered the *convective* heat release rate, that is, 90 % of the heat from the fire is lofted into the plume. The remaining 10 % of the heat released is assumed to be radiated away, and plays no role in the model. The particulate mass flux was determined by multiplying the mass of oil consumed by the measured smoke yield of ANS crude (11.6 %), divided by the burn time.

Atmospheric temperatures, wind speeds and wind directions were measured with a weather station suspended from a tethered mini-blimp, deployed just after the burns were completed. However, the wind speed for the second burn was too high to use the mini-blimp, and the temperature profile was taken from a helicopter, the wind speed and direction estimated from the flight log of the airplane and ground weather stations.

**Fig. 32:**
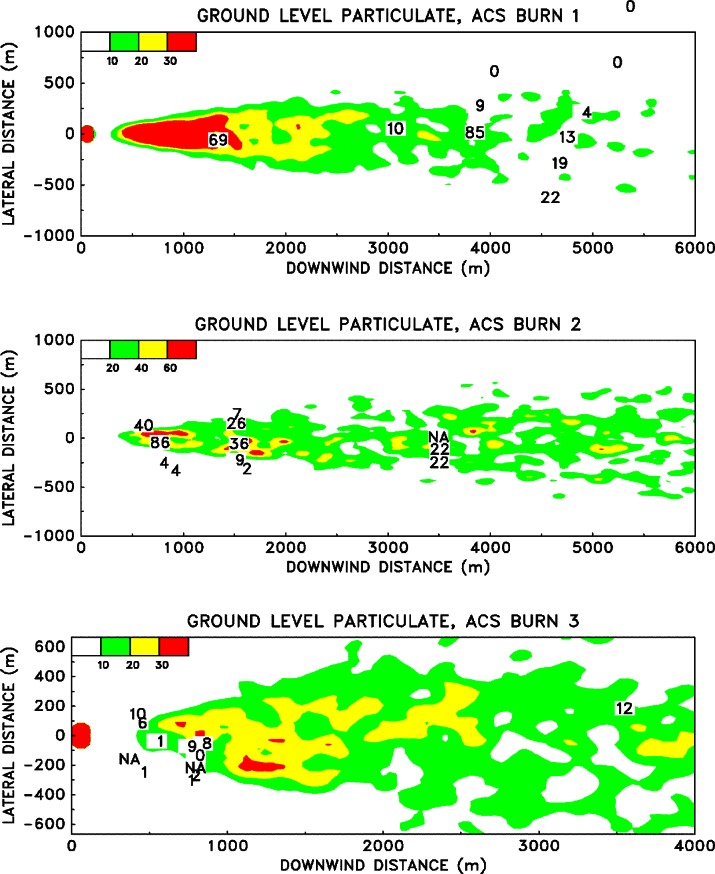
ALOFT predictions of ground level particulate concentrations (shaded contours) along side the actual time-averaged RAM data for the three ACS Emulsion Burns. All concentrations are given in units of µg/m^3^.

The first burn lasted about 47 min, during which time the area of burning surface varied from practically zero to the full area of the boom plus spillover. In all, nine “pulses” of several minutes in duration occurred. Due to the unsteady burning, the downwind instruments detected a number of “hits” due to the fact that the smoke generated when the fire was small was not lofted very high. The first plot of Fig. 32 summarizes the model simulation of Burn 1, showing the model prediction of ground level particulate concentration versus the actual measurements made in the field. The field measurements were averaged over the time of the burn. Neither the model predictions nor the RAM data is uniform in space or in time, due in part to random fluctuations in wind direction, convective cells which are not accounted for in the model, small terrain effects, and unsteady burning of the fuel. Nevertheless, the time-averaged model predictions and field measurements agree to within the uncertainty of the measured fire and meteorological conditions [58], showing particulate concentrations ranging from 0 µg/^3^ to 80 µg/m^3^ along the narrow path over which the plume is lofted. In addition to ground level instruments, a small airplane flew in the vicinity of the plume and recorded plume positions at various times, as well as photographed the burn site and the plume. According to flight track data, the plume top rose to a height of about 550 m to 600 m, in agreement with model predictions.

**Fig. 33:**
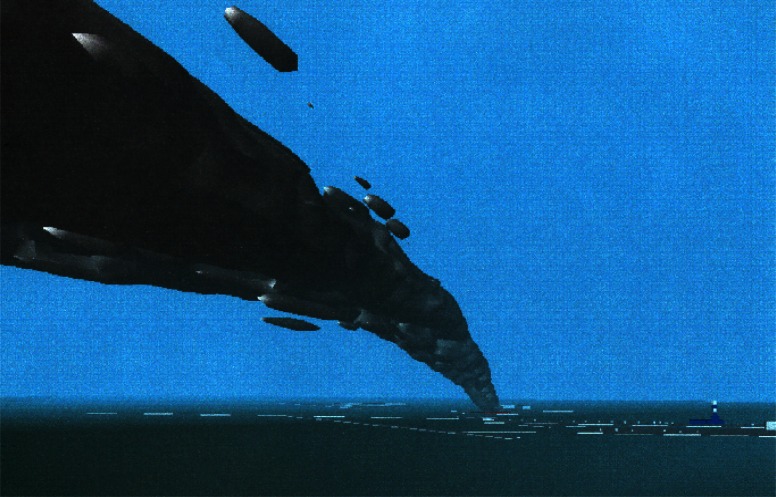
Downwind view of the simulated smoke plume from the second ACS emulsion burn, Prudhoe Bay, September 1994.

The second burn was conducted for two reasons. First, it provided a control with which to compare the two emulsion burns. Second, it served as a test case to compare to the numerical model since the smoke yield and heat release rate from a large pool fire of unweathered, unemulsified oil are relatively well known from previous laboratory and mesoscale experiments [28, 69]. The second plot of Fig. 32 summarizes the model prediction versus field measurements for Burn 2. Of particular interest in this burn was the presence of a thermal inversion at about 300 m. This inversion layer restricted the plume to a maximum height of about 400 m, and again this altitude was verified by the accompanying flight track recorder from the airplane. The wind variability was less than that recorded for the first or the third burn, yielding a plume which retained its basic shape and structure for about 10 km. Figure 33 presents a downwind view of the simulated smoke plume.

Even though the fuel for the third burn resembled that of the first, the burn was much steadier than the first. This probably was due to a slight modification in the application of the emulsion breaker. In any event, weather conditions on the day of the third burn (September 11) were so foggy that the helicopter, which had been used on previous days to place instruments in the field, was grounded. The wind was from the north, blowing directly over a river bed, but shifting about 10° per hour. Because of the bad terrain and visibility, it was decided to deploy the instruments in the near field, all within a kilometer of the pit except for one which was sent with a monitor further afield. The third plot of Fig. 32 summarizes the numerical prediction and field measurements from the third burn.

#### 5.3.3 Mesoscale Diesel Fuel Burns, Mobile, Alabama

**Fig. 34:**
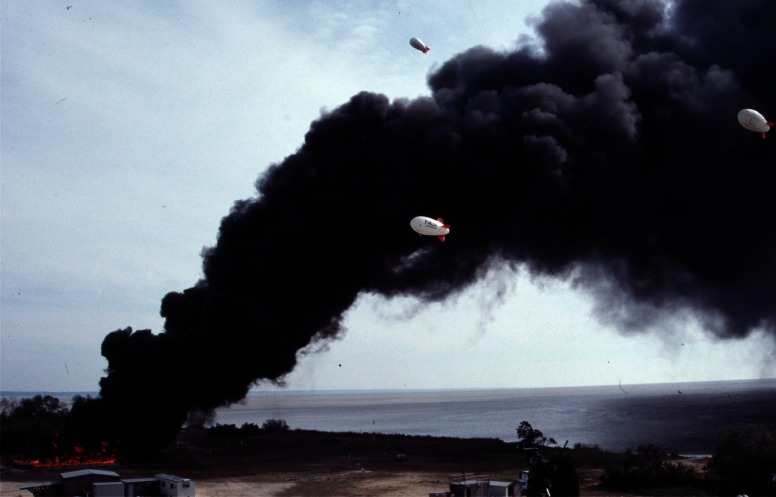
Photograph of a diesel fuel burn at the U.S. Coast Guard Fire and Safety Test Detachment, Mobile, Alabama.

Three mesoscale burns of number 2 diesel fuel were conducted by NIST at the US Coast Guard Fire and Safety Test Detachment facility on Little Sand Island in Mobile Bay, Alabama in October 1994 [71]. The burns were conducted in a 15.2 m square by 0.61 m deep steel burn pan. Water filled about 0.5 m of the pan, and diesel fuel was added to fill the rest. The number 2 diesel fuel was obtained from a commercial vendor. Figure 34 is a photograph of one of the burns. Table 7 summarizes the relevant information for each burn. Note that the first burn was conducted with a fire resistant boom forming one edge of the burn area, thus its burn area is slightly smaller than the second two burns. This is the reason for the slightly longer burn time and slightly lower heat release rate. The smoke yield for number 2 diesel had been measured at a previous burn series in Mobile to be about 14 % [66].

Only ground level meteorological information was available from two stations on the island. The wind speeds and fluctuations during the burn of October 23 correspond to Pasquill stability class A or B, while the conditions of October 26 correspond to class C. The mixing layer depths were determined from the analysis of lidar data that will be described below. Following is a description of each burn, plus an analysis of the predicted and measured plume concentrations.

The first burn was ignited in the afternoon on the 23rd of October. The winds were calm (1 m/s to 2 m/s), and as a result the smoke plume rose 2 km into the atmosphere and mixed into a cloud layer. There was no mixing of the plume down to the ground. The combination of wind speed and mixing layer depth for this burn lies at the very fringe of the parameter space for which the ALOFT model was designed. The meteorological conditions on the day the second and third burns were conducted were within the parameter space for which the ALOFT model was designed. On this day, the wind was blowing from the north, and the smoke plumes from both burns lofted over the western shore of Mobile Bay and out into the Gulf of Mexico. A team from SRI, International, of Menlo Park, California, performed airborne lidar measurements of the smoke plumes [72]. The instrument was flown above the smoke plume, generating cross-sectional images of the plume in vertical planes *perpendicular* to the direction of the wind at various distances downwind of the fire. For the morning burn, the depth of the mixing layer was about 450 m, and in the afternoon it had risen to about 700 m. Although a temperature sounding could not be obtained on that day, it is clear from the lidar images of the smoke plume that the top of the mixing layer at both times of the day corresponded to a shift in wind direction, and probably a temperature inversion. The wind was blowing out of the north at ground level, but apparently shifted to become northeasterly above the mixing layer top. This wind shear is very noticeable due to the fact that most of the smoke particulate is concentrated in that narrow band. The smoke that mixes down to the surface does so at the interface between land and water, in a process known as fumigation.

**Fig. 35:**
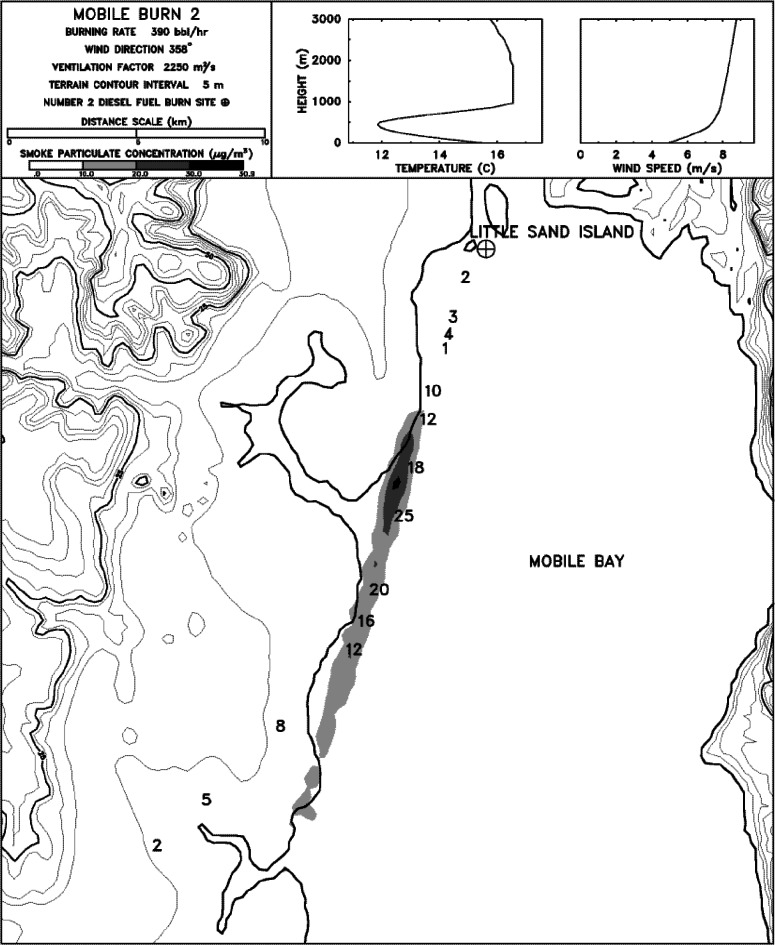
ALOFT predictions of ground level particulate concentration for the morning burn of October 26, 1994, in Mobile Bay. The shaded contours represent model predictions, the numbers represent near ground peak values (µg/m^3^) of the quantified lidar signatures for each pass of the aircraft. The ventilation factor is the depth of the mixing layer multiplied by the wind speed, and is used as a rough indicator of atmospheric stability.

**Fig. 36:**
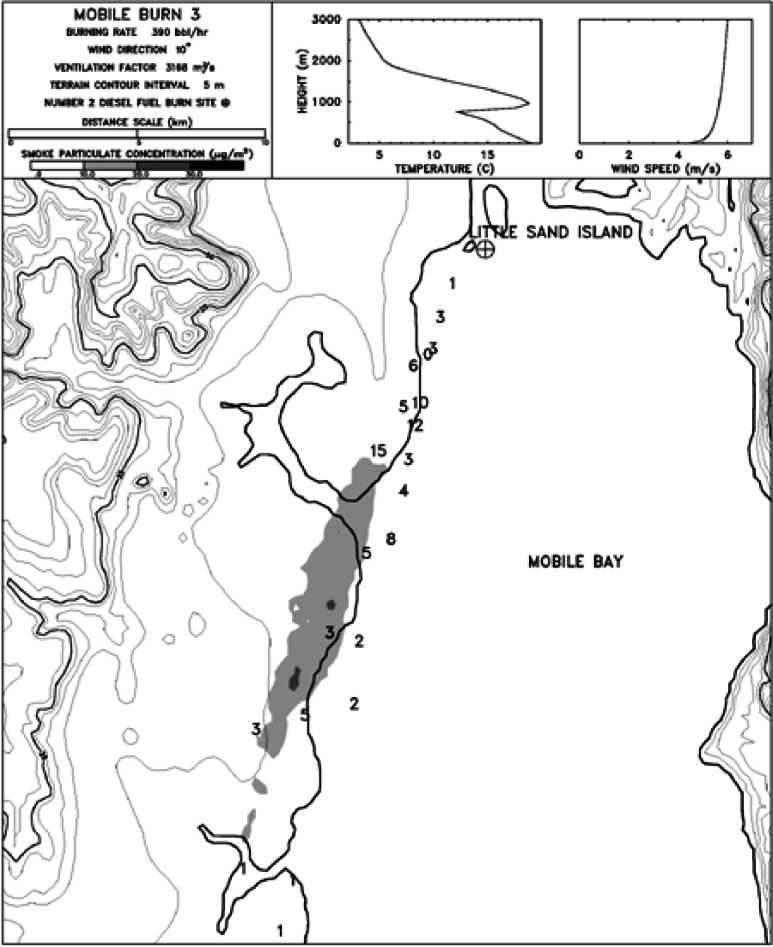
ALOFT predictions of ground level particulate concentration for the afternoon burn of October 26, 1994, in Mobile Bay. The shaded contours represent model predictions, the numbers represent near ground peak values (µg/m^3^) of the quantified lidar signatures for each pass of the aircraft. The ventilation factor is the depth of the mixing layer multiplied by the wind speed, and is used as a rough indicator of atmospheric stability.

The ALOFT model was run to simulate the second and third burn using a non-linear temperature profile. The ground level concentration predicted by the calculations is lower than that predicted by the linear profile correlations. The reason for the difference is that the plume in the non-linear case penetrates the inversion layer, and there is less mixing of the particulate back to the surface. The linear correlations do not account for this effect. Indeed, the unlimited vertical mixing assumption made in deriving the linear correlation is the main reason for its conservative bias. Figures 35 and 36 summarize the ground level prediction of smoke particulate concentration from the model, along with the maximum values of the lidar measurements for each pass of the aircraft above the plume. The particulate concentrations are derived from the lidar signatures by assuming constant backscatter-to-density and extinction-to-density ratios. The latter quantity was derived by the University of Washington team for their analysis of the Newfoundland lidar data [68]. The model prediction of the location of the peak concentration for burn 2 agrees well with the lidar measurement. For burn 3, the model appears to overpredict the distance of peak concentration. In both cases, the magnitude of the ground level concentration is in agreement with the lidar measurements. Again, “agreement” infers that the model prediction is within the uncertainty range established by the uncertainty of the meteorological and fire conditions, plus the uncertainty of the lidar quantification.

As in the analysis of the Newfoundland data, it is impossible to replicate with the *steady-state* model every meteorological detail reflected in the *instantaneous* lidar measurements. Instead, it is assumed that the wind fluctuation and vertical convective motion are random processes. In this way, the plume structure and the local meteorology can be described in sufficient detail to produce predictions in the neighborhood of the measured concentrations.

#### 5.3.4 Discussion of Field Experiments

Small and large scale experiments play two key roles in the modeling process. First, measurements of the fires furnish the heat release rate and emission factors for the combustion products. The model does not predict these quantities. Second, the experimentally measured downwind concentrations of smoke particulate can be compared against the model predictions to determine their accuracy and to assess whether new physical phenomena should be included in the model, such as radiative heating, unsteady burning, and smaller-scale atmospheric motion. The decision to include or exclude these effects is based on how well the model performs in comparison to the results of the experiments. For the three experiments discussed in this report—NOBE, the ACS Emulsion Burns, and the Mobile burns—none of the observed secondary effects was important enough to merit a change of the numerical algorithm. It should be noted that the large scale experiments are *not* used to calibrate the model. That is, the processes governing the entrainment, mixing and dispersion of combustion products do not rely on empirical parameters.

The results of the experiments presented here increase the confidence in the numerical predictions of plume structure, trajectory and composition. The comparison of predicted versus measured particulate concentration is very encouraging, given the uncertainties in the fire and weather characterization. In fact, the model predictions were based on very limited meteorological information—wind speed, wind variation and temperature stratification only. This is important for two reasons. First, local meteorological data for regions of interest is often very limited. Second, if the numerical model is to be used effectively for a wide variety of conditions, it must not depend on empirical input parameters tuned for a particular situation.

As far as the field measurement techniques are concerned, these experiments have provided a wealth of information on how to monitor emissions from large burns. Unlike conventional air monitoring where the source, such as a power plant, is expected to generate pollutants over a long period of time, an in situ burn will typically last a few hours. High volume samplers are difficult to position and cannot collect enough particulate in that short period of time, hence the need for reliable, portable real-time aerosol monitors. For the purpose of model verification, lidar measurements have the most potential because they can capture the overall plume structure rather than sparse points. The drawbacks of the lidar are that it is expensive, and the measurements are difficult to quantify.

Of all the experiments discussed within this paper, the smoke plumes from the Mobile burns, although of short duration, are most representative of those that can be expected from an actual in situ burn for two reasons. First the burning rate of 64000 L/h is probably a reasonable rate to expect from an actual burn. It has been estimated that a 150 m fire boom towed in a U-shape configuration could easily provide enough oil area to sustain a burn eliminating about 114000 L/h [73]. Second, the experiments were conducted in a coastal environment, thus the atmospheric conditions represented by the lidar images are very typical of what one can expect in the event of a near-shore in situ burn. The results of both the modeling effort and the lidar measurements showed that even though an inversion layer was present, the plume penetrated it, and as a result less smoke was mixed back to the surface. The plume will not always penetrate an inversion layer, and in those instances ground level concentrations could be higher.

In summary, peak concentrations of ground level smoke particulate for all the burns discussed above never exceeded 100 µg/m^3^ (averaged over the time of the burn) beyond a few hundred meters from the fire, and in most cases were well below that level. It should be emphasized, however, that these experiments were conducted in reasonably good weather conditions, and in each instance, complex terrain was not a factor.

## 6. Guidelines for in situ Burning

To facilitate the approval of in situ burning as an oil spill response method, the Alaska Department of Environmental Conservation sought assistance from NIST to use its newly developed ALOFT model for smoke plume trajectory to help develop guidelines for approval of intentional burning of spills. Two in situ burning scenarios were developed by NIST: one representing the burning of Cook Inlet crude oil in the Cook Inlet region and the other North Slope crude oil in the North Slope region.

Laboratory tests were performed at NIST to measure the burning and smoke yield properties of the two crude oils [28]. Based on the results of larger scale burns available at that time, the laboratory measurements were extrapolated to determine the smoke yield from large burns of the two crude oils. For the Cook Inlet crude the smoke yields were estimated to be about 9 % for the Cook Inlet crude oil and 12 % for the North Slope crude oil. A matrix of 28 different burning conditions varying oil type, burn area, location, season of the year, wind speed, and atmospheric temperature profiles was constructed [11]. For each case, 1 h average ground level smoke particulate concentrations were determined as a function of distance from the burn.

Air quality regulations in Alaska do not contain any specific information about levels of concern for smoke particulate from oil fires. However, a 150 µg/m^3^ particulate concentration averaged over 24 h is regarded generally as an upper limit for acceptable air quality.

For the range of parameters used, the calculations showed that particulate concentrations found at the ground downwind of oil spill burns will not exceed 150 µg/m^3^ beyond 5 km, nor outside of a strip approximately 1 km wide along the centerline of the plume trajectory. In one-third of the cases, the concentrations fell below 150 µg/m^3^ in less than 1 km from the burn. This work provided new insight into the probable areas of concern downwind of oil spill burns. The results showed that this distance was far less than previous thought.

In 1994, the State of Alaska used the results of this NIST research as a basis for revision to their guidelines for approval of in situ burning [74]. In the acceptability section the guidelines state,

Based upon the finding of the NIST report, “Smoke Plume Trajectory from In Situ Burning of Crude Oil in Alaska,” the ARRT [Alaska Regional Response Team] has set a worse case, conservative downwind distance of 10 kilometers or approximately 6 miles as the primary value for “a safe distance” to conduct burning operations away from the human population… This distance may be modified (decreased or increased) after evaluating spill specific data such as location of spill, type of oil, and stability class of current meteorological conditions. If the burn involves either Cook Inlet or North Slope Crude and is located on the North Slope or in South Central Alaska, i.e., Cook Inlet/Prince William Sound, values from Table 7 [Burn Scenarios] of the NIST report, which presents a summary of smoke trajectory runs, may be utilized with a safety factor of 2X. Table 7 is included as an attachment to this review checklist.

**Fig. 37:**
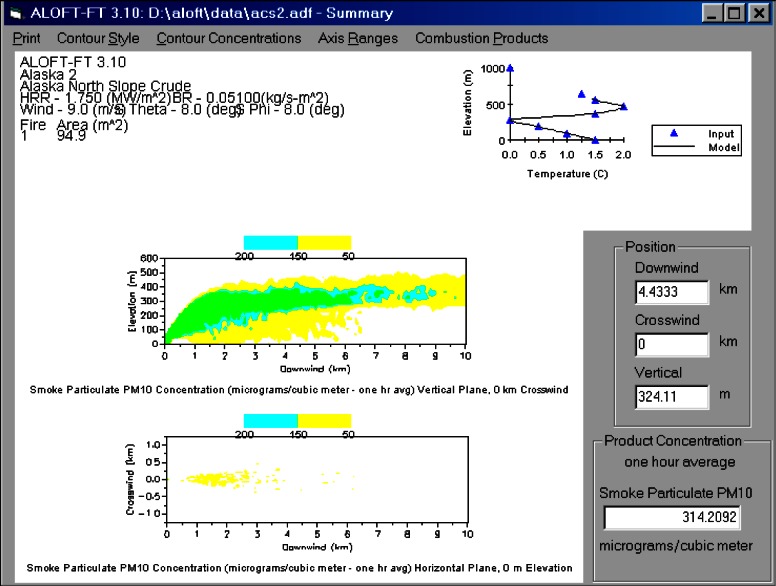
Example output screen from the NIST ALOFT-FT personal computer software used to quantify down wind particulate concentrations.

In order to put the capabilities of performing smoke trajectory calculations in the hands of responders for the purpose of assessing the acceptability of initiating in situ burning considering specific conditions at a site, NIST developed the ALOFT-FT smoke plume trajectory software for personal computers [75, 76]. This software produces trajectory predictions and downwind particulate concentrations within the uncertainty of the computations performed with more powerful computers at NIST, but is capable of being run on portable computers in the field. A user-friendly interface was developed to allow users to input available data from site measurements or simply observations so that the calculation could be as specific to the incident as possible. Responders have also found the graphic output (Fig. 37) provided by the model useful in explaining the findings of the calculations to local authorities for approval for intentional burning.

Results from the ALOFT-FT model were used by local officials in the decision to intentionally burn fuel onboard the freighter, *New Carissa* grounded in Coos Bay Oregon in February 1999. Burning was the only response option feasible to reduce the potential for a disastrous oil spill from the imminent breakup of the ship. The ALOFT-FT model was cited by the on-scene scientific advisors as providing the timely and critical information about the impact of burning on air quality.

Equally important to the quality of the computations was the quality and clarity of the graphic presentation of the results. The ALOFT-FT software provided information on the smoke plume trajectory and downwind concentrations in a manner that could be easily understood by local officials and public interest groups involved with the incident. The combined visual presentation of technical results provided by ALOFT-FT, the long history of verification testing, and the reputation of NIST as a source of high quality measurement and prediction technology provided the confidence for approval of intentional burning. This incident is the first time that intentional burning received wide spread publicity in the United States as a spill mitigation technique. Removing oil from the ship by burning helped to prevent millions of dollars of shoreline clean-up costs that would have occurred as the grounded vessel, battered by waves ruptured and split into two pieces shortly after the burns.

## 7. Conclusion

NIST measurement and prediction efforts have played a major role in establishing in situ burning as an oil spill response method for use in the United States to minimize the pollution from oil spills. The better understanding of oil spill burning and the consequences produced by the NIST research enabled guidelines to be established whereby in situ burning is now considered to be a primary oil spill response technology. Burning is no longer regarded as an oil spill response method of last resort.

Important data has been generated to quantify the smoke particulate in large fire plumes. Methods have been developed to reliably predict the downwind concentrations of particulate transported by wind blown fire plumes. Tools have been developed to make this information accessible and usable by the fire and oil spill response communities.

## Figures and Tables

**Table 1:** Summary of smoke emission data for 40 and 60 cm diameter Prudhoe crude pool fires

Property	Test NW33 40 cm pool	Test NW34 40 cm pool	Test NW35 60 cm pool	Test NW36 60 cm pool
d*Q*/d*t*, (kW)	63	61	173	178
d*m*/d*t*, g/s	1.81	1.75		
*ε*_1_	0.107	0.096		
*ε*_2_	0.098	0.079	0.080	0.090
*φ* (CO_2_), vol. fraction	1.5 × 10^−3^	1.8 × 10^−3^	5.2 × 10^−3^	5.3 × 10^−3^
*φ* (CO), vol. raction			0.32 × 10^−3^	0.32 × 10^−3^
*K*_s_, (m^2^/g)	8.95	10.00	8.80	8.64

**Table 2:** Comparison of flux method to carbon balance field method

Test	Flux method	Carbon balance field system
1	0.129	0.145
2	0.115	0.133
3	0.130	0.144
Average	0.125	0.141
Standard deviation	0.008	0.007

**Table 3:** Mean PAH concentration for Alberta sweet crude oil smoke samples

No.^b^	PAH	PAH concn,^d^ µg/g							
		Collected at 100 °C				Collected at 25 °C			
		H2 #1^c^	H2 #2	H4 #1	H4 #2	C5 #1^c^	C5 #2	C7 #1	C7 #2
	acenaphthylene			3	2			35	59
	acenaphthene			ND	ND			ND	1
	fluorene			4	3			130	140
1.	phenanthrene	31^e^	21^e^			910	1040		
				95^f^	58^f^			1220^f^	1220^f^
2.	anthracene	ND	ND			260	290		
6.	fluoranthene	47	37	210	130	700	730	740	720
7.	acephenanthrylene	22	22						
8.	pyrene	65	49	260	150	900	840	800	780
9.	benzo[ghi]fluoranthene	69	65			240	220		
10.	cyclopenta[cd]pyrene	24	110			540	540		
11.	benz[a]anthracene	63	87	88	180	220	220	240	220
12.	chrysene/triphenylene	71	87	95	190	220	230	220	220
13.	benzofluoranthenes	190	350	490	520	380	390	420	410
15.	benzo[e]pyrene	66	110	92	220	120	120	180	170
16.	benzo[a]pyrene	110	200	73	190	240	180	210	200
17.	perylene	19	38	18	52	42	41	45	47
18.	indeno[1,2,3-cd]pyrene	110	220	250	750	190	190	540	530
19.	benzo[ghi]perylene	130	240	87	240	210	210	180	180
	Total PAH^g^	1020	1636	1760	2680	5172	5240	4960	4897

a Samples H2 and C5 analyzed by NIST, H4, and C7 analyzed by EC.

b Peak identification numbers for Fig. 23.

c Micrograms of PAH per gram of smoke; uncertainty estimated to be ±10 % of the reported concentration for samples H2 and C5.

d ND, not detected.

e Concentration calculated by using phenanthrene-*d*_10_ as the internal standard; all other concentrations computed on the basis of 1-*n*-butylpyrene as the internal standard for samples H2.

f This number represents the sum of phenanthrene and anthracene.

g Only PAHs common to both analyses (NIST and EC) are included in the sum.

**Table 4:** Concentration of PAHs in Alberta sweet crude oil and its burn residue^a^

	µg of PAH/g of sample				
	Crude oil	Crude oil	Residue D	Residue Z	Residue Y
	Q	S	2 mm layer	5 mm layer	10 mm layer
acenaphthylene	13		54	26	
acenaphthene	57		15	10	
fluorene	59		47	35	
2-methylfluorene	150		110	160	
phenanthrene	150	150	140	130	120
methylphenanthrenes		370			330
dimethylphenanthrenes		500			520
anthracene	11		19	13	
fluoranthene	6		22	11	
pyrene	17		30	25	
1-methylpyrene	39		16	19	
benzo[ghi]fluoranthene			1	2	
chrysene/triphenylene	30		24	34	
benzo[b]fluoranthene	4		7	2	
benzo[e]pyrene	5		6	6	
benzo[a]pyrene			4	3	
2-methylcholanthrene	3		3	3	
benzo[ghi]perylene			2		
Total PAH	540	1020	500	480	970

a Samples S and Y were analyzed at at the NIST and samples D, Z, and Q were analyzed at EC.

**Table 5:** Comparison of PAH content of crude oil, oil residue, and smoke (vapor and particulate) and wood per gram of fuel consumed

µg of PAH/g of fuel consumed							
Sample/layer thickness	3 rings	3 rings/methyl	4 rings	4 rings/methyl	5 or more	5 or more rings/methyl	total
Alberta sweet	290	1020	53	39	9	3	1440
Residue							
2 mm	120	470	35	7	10	1	640
3 mm							380^a^
5 mm	50	240	17	5	4	1	320
10 mm							230^a^
Smoke^b^							
2 mm	62 (120)^c^	2	69	2	71		210 (330)^c^
3 mm	100 (190)	2	130	3	110		350 (440)
5 mm	90 (230)	8	170	4	120		390 (530)
10 mm	180 (320)	8	260	4	180		630 (770)
Wood birch^d^	7	2	8	0.2	3		20

a The overall estimates for the 3 mm and 10 mm layers are based on the results for the 2 mm and 5 mm layers and the measured residue fraction.

b In computing the smoke results using Table 3, the missing PAH values such as benzo[a]fluoranthene for the 2 mm layer thickness are estimated based on related entries in Table 3 (the 3 mm test in the case of benzo[a]fluoranthene.

c The values in parentheses include acenaphthylene.

d See Ref. [20].

**Table 6:** Summary of the ACS mesoscale emulsion burns

	Burn 1	Burn 2	Burn 3
Date	Sept. 8	Sept. 10	Sept. 11
Volume of emulsion (m^3^)	7.7	12.2	16.6
Percent oil	50 %	100 %	60 %
Oil mass (kg)	3768	10827	6545
Oil removal efficiency	97.3 %	98.4 %	96.7 %
Burn time (min)	47	40	45
Estimated heat release rate (MW)	55	186	98
Estimated particulate mass flux (kg/s)	0.15	0.51	0.27

**Table 7:** Summary of the mobile burn series, October 1994

	Burn 1	Burn 2	Burn 3
Date	Oct. 23	Oct. 26	Oct. 26
Burn area (m^2^)	199	231	231
Fuel volume (m^3^)	17.1	17.1	17.1
Burn time (min)	19	15	15
Burning rate (kg/s/m^2^)	0.063	0.067	0.067
Total heat release rate (MW)	484	602	598
Particulate mass flux (kg/s)	1.75	2.18	2.18
Ground wind speed (m/s)	1.6 ± 0.8	5.1 ± 1.7	4.7 ± 1.5
Mixing layer depth (m)	2000	450	700

## Footnotes

1 Certain commercial equipment, instruments, or materials are identified in this paper to foster understanding. Such identification does not imply recommendation or endorsement by the National Institute of Standards and Technology, nor does it imply that the materials or equipment identified are necessarily the best available for the purpose.
